# Benchmarking Density Functional Approximations for Diamagnetic and Paramagnetic Molecules in Nonuniform Magnetic Fields

**DOI:** 10.1021/acs.jctc.0c01222

**Published:** 2021-02-12

**Authors:** Sangita Sen, Erik I. Tellgren

**Affiliations:** † Department of Chemical Sciences, Indian Institute of Science, Education and Research, Kolkata 741246, India; ‡ Hylleraas Centre for Quantum Molecular Sciences, Department of Chemistry, University of Oslo, P.O. Box 1033, Blindern, N-0315 Oslo, Norway

## Abstract

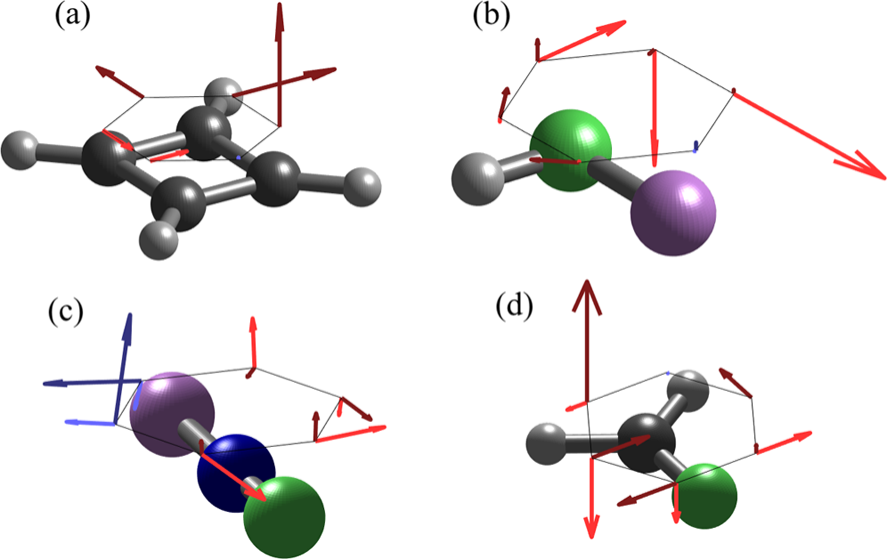

In this article, correlated studies on a test set of 36 small molecules are carried out with both wavefunction (HF, MP2, CCSD) and density functional (LDA, KT3, cTPSS, cM06-L) methods. The effect of correlation on exotic response properties such as molecular electronic anapole susceptibilities is studied and the performance of the various density functional approximations are benchmarked against CCSD and/or MP2. Atoms and molecules are traditionally classified into “diamagnetic” and “paramagnetic” based on their isotropic response to uniform magnetic fields. However, in this article, we propose a more fine-grained classification of molecular systems on the basis of their response to generally nonuniform magnetic fields. The relation of orientation to different qualitative responses is also considered.

## 1 Introduction

Magnetic field effects pose unique challenges for quantum chemistry. Although the calculation of particular properties, most notably magnetic dipoles, nuclear shielding constants, and current densities induced by uniform magnetic fields, is nowadays routine, many other aspects have been subject to relatively few systematic studies, if any at all. Higher-order magnetic response and response to nonuniform magnetic fields are examples of this.^1−4^ Moreover, nonperturbative effects of strong magnetic fields alter the normal chemistry of small molecules, giving rise to an exotic and largely unexplored strong field chemistry.^5−7^ These challenges appear at all levels of theory, as for example, time-reversal and spin symmetry and other built in adaptations to zero-field settings need to be reconsidered. In density functional theory, in particular, an additional aspect is that magnetic field effects are formally beyond the scope of the standard mathematical formulation, necessitating extensions.^8−11^ Yet, the practically available density functional methods have almost exclusively been developed as heuristic approximations with the standard formulation, and approximations that properly incorporate magnetic fields are not yet mature enough to be practically useful.^12^ Insofar as available density functional methods produce useful estimates of magnetic field effects, there is a high risk due to error cancellations specific to the most common magnetic properties. For example, some functionals have been fitted to magnetizabilities or nuclear shielding constants.^13^ In the present work, we explore properties related to magnetic field gradients using several available density functional methods, including meta-GGAs that have recently emerged as particularly promising candidates for magnetic field effects, and compare to results at the second-order Møller–Plesset (MP2) and coupled-cluster singles and doubles levels (CCSD).

Quantum chemical computations of magnetic properties almost always rely on the assumption of weak-fields enabling formulations based on perturbation theory. However, for a higher-order magnetic response, in particular when London atomic orbitals (LAOs) are employed to enforce gauge-origin invariance and accelerate basis set convergence,^14−17^ the perturbative approach becomes increasingly more difficult. Ordinary Gaussian basis sets require very large basis sets for gauge-origin invariance.^1,3,4,18,19^ In this study, we have used LAOs in combination with a nonperturbative (finite field) approach. Integral evaluation for the LAOs, which are plane-wave/Gaussian hybrid functions,^20,21^ has been implemented in the London program^20,22^ and has been employed in the finite field computation of magnetic properties^7,12,20,23−25^ earlier. Since the introduction of the magnetic field in the Hamiltonian only requires a modification of the one-electron part, no additional implementation is necessary for extension to post-Hartree–Fock methods. A nonperturbative approach also opens up the possibility of studying strong magnetic fields competing with the Coulomb forces and has led to the discovery of nonperturbative transition from closed-shell para- to diamagnetism^26^ and a new bonding mechanism^6,7,27^ in very strong magnetic fields.

In this work, we study the effect of correlation on anapole moments arising from induced orbital currents for a set of 36 closed-shell molecules subject to transverse magnetic field gradients. We have benchmarked LDA (SVN5),^28^ KT3,^13^ cTPSS,^29^ and cM06-L^30,31^ functionals against CCSD and/or MP2. An earlier study^32^ to assess the effects of correlation was inconclusive. CCSD results were reported in the aug-cc-pVDZ basis set, which is too small for accurate representation of such high-order properties. Moreover, the relative quality of the density functional approximations studied (KT3,^13^ B3LYP,^33^ CAMB3LYP^34^) could not be established. Basis set convergence of the anapole susceptibility values was well-studied and MODENA basis sets were proposed to be superior to Dunning’s basis sets. Previous studies of density functional approximations for magnetic properties like magnetizabilities and NMR shielding constants^12,35,36^ have indicated that meta-GGA functionals, in particular cTPSS, are promising candidates for capturing even exotic magnetic effects very far from the domain these functionals were explicitly constructed for. Remarkably, it was also found that the errors for the paramagnetic closed-shell molecules were an order of magnitude higher for all of the methods studied except for cTPSS (current TPSS). The present work investigates whether this trend holds for more exotic magnetic properties like anapole susceptibilities, which have not been considered thus far.

Because paramagnetic systems typically exhibit stronger correlation effects than diamagnetic systems, we will generalize below these concepts to allow for nonuniform magnetic fields. Historically, the discovery of diamagnetism is credited to Anton Brugmans who observed in 1778 that bismuth was repelled by magnetic fields.^37^ William Whewell suggested the terminology *diamagnetic* for materials repelled by a magnetic field and *paramagnetic* for those attracted by it and Faraday adopted this.^38^ The quantum picture of atomic and molecular magnetism was established with van Vleck’s theory of paramagnetism and crystal field theory for solid-state magnetism, Dorfman’s corresponding theory for metals, Pauli’s work including the derivation of temperature independent paramagnetism, and Landau’s quantum theory of diamagnetism. Here, we have bypassed the vast field of ferromagnetism, since this article is not concerned with it.

In modern terms, dia- and paramagnetism are understood in terms of whether the (second-order) response of the energy to a uniform field is positive or negative. In the mathematical literature, this distinction has also been used for arbitrary, nonuniform magnetic fields.^39,40^ Because a uniform field is a three-dimensional vector, the second-order magnetic susceptibility is a 3 × 3 tensor. Taking into account a sign convention, clear-cut examples of diamagnetism (or paramagnetism) occur when this tensor only has negative (or positive) eigenvalues. However, the tensor may also have both positive and negative eigenvalues, corresponding to the decreasing energy for some, but not all, magnetic field components. Conventionally, the sign of its isotropic value decides the classification of the molecule as dia- or paramagnetic. For example, the BH molecule is found to have a diamagnetic response to fields parallel to the chemical bond and a paramagnetic response to fields perpendicular to it. In this case, the isotropic magnetizability turns out to be paramagnetic as well leading to the classification of BH as a closed-shell paramagnetic molecule. However, a molecule such as square C_4_H_4_ shows a weak paramagnetic response to fields perpendicular to the molecular plane but an overall diamagnetic response.

The magnetic susceptibilities related to inhomogeneities in the magnetic field such as the anapole susceptibilities are independent of the magnetizabilities and may in some cases oppose the effects of the uniform component of the magnetic field. In our opinion, a classification should also encompass molecular response to nonuniform magnetic fields in general, as far as possible. In what follows, we propose a simple classification of magnetic response to nonuniform fields. The response of the electrons to inhomogeneities in the external magnetic field arises from both orbital effects and spin effects. Among the few studies of these effects is the work by Lazzeretti and co-workers on a perturbative formalism for the orbital response due to field gradients^41,42^ and some other studies at the Hückel-level,^43^ Hartree–Fock level,^1,3,4,18,19^ and correlated levels.^32,44^ While spin effects are certainly important and in most cases the dominant effect,^25,45^ this article is only concerned with orbital effects in closed-shell molecules. Further exploratory studies are planned but beyond the present scope.

The response to (transverse) magnetic field gradients may be quantified by the anapole moments,^46^ which couple linearly to the curl of the magnetic field.^47−52^ They may be considered to arise from the meridional currents in a toroidal charge distribution. They are antisymmetric under both spatial inversion and time-reversal. Nuclear anapole moments are studied by physicists^53,54^ in connection with parity violation with the first experimental evidence coming from measurements on the Cs atom.^55−57^ Experiments for measuring permanent and induced electronic anapole moments have been suggested.^49,58,59^ However, only special structures such as molecular nanotoroids,^43,60^ ferroelectric nanostructures,^61,62^ ferromagnetic structures,^63^ and Dy clusters (single-molecule magnets)^64−66^ are expected to have permanent anapole moments. Anapole moments in metamaterials have also been observed with potential application in sensors.^50,52,67^ On the other hand, induced anapole moments easily arise in molecules placed in external nonuniform fields and we can compute the corresponding susceptibilities. Both toroidal spin and/or orbital currents can give rise to anapole moments. Induced anapolar current densities in conjugated cyclic acetylenes^60^ and some small molecules^58^ have been studied. Spin and orbital contributions to anapole moments have been analyzed in a simple analytical model of diatomics^59,68^ and also using nonperturbative General Hartree–Fock theory.^25^ The orbital contributions have been estimated by both perturbative approaches^1,3,4,18,19^ and nonperturbative approaches.^23^ Faglioni et al.^1^ have derived the perturbative expressions for induced anapole moments.

The outline of the article is as follows. In Section 2, we define the Hamiltonian and the properties relevant to our study. Section 3.2 discusses our proposed classification of molecules. Section 3 presents our results on the effect of correlation on the anapole susceptibilities and the relative performance of the various density functional approximations. Finally, we conclude with the summary in Section 4.

## 2 Hamiltonian and Properties

In this study, the nonuniform magnetic field has the form
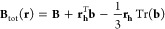
1where **B** is a uniform (position independent) component, **b** is a 3 × 3 matrix defining the field gradients, and **r**_**h**_ = **r** – **h** is the position relative to some reference point **h**. This form may be viewed as arising from a truncation of a Taylor expansion of a general magnetic field around **r** = **h** of linear order. The corresponding vector potential can be written as
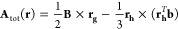
2where **r**_**g**_ = **r** – **g**, **g** being the gauge origin. One can show that **B**_tot_ = ∇ × **A**_tot_ and that the magnetic field is divergence free, ∇ · **B**_tot_ = 0. The symmetric part, **b** = **b**^*T*^, can be set to zero and we focus on the antisymmetric part *C*_α_ = ϵ_αβγ_*b*_βγ_ of the matrix **b**. We can then write
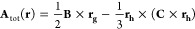
3
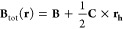
4Furthermore, the antisymmetric part of **b** equals the curl of the magnetic field, ∇ × **B**_tot_ = **C**, which is taken to be position independent.

The nonrelativistic Schrödinger–Pauli Hamiltonian is given by
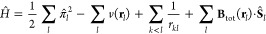
5where **π̂**_*l*_ = −*i*∇_*l*_ + **A**_tot_(**r**_*l*_) is the mechanical momentum operator. Properties can be alternately viewed as expectation values ⟨Ψ|Ω̂|Ψ⟩ or as derivatives of the energy *E* = ⟨Ψ|*Ĥ*|Ψ⟩ related to terms in a Taylor expansion. In this study, we ignore the spin-Zeeman term and thereby the spin-breaking induced by the nonuniform part of the magnetic field. The first-order orbital angular moment
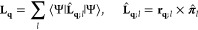
6is with respect to an arbitrary reference point, **q**. Given the form of the magnetic vector potential above, it is **L**_**g**_, with the reference point at the gauge origin that is the relevant magnetic dipole moment. The orbital anapole moment is similarly given by
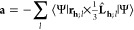
7

Recalling that the current density can be obtained as the functional derivative **j** = δ*E*/δ**A**_tot_, we can also identify the magnetic orbital dipole moment and anapole moment with linear and quadratic moments of the current density
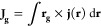
8
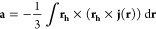
9

We note that the energy *E* as well as expectation value properties like **J**_**g**_ and **a** can be obtained directly as functions of **B** and **C**. We can thus define second-order properties from a Taylor expansion of the energy
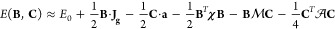
10where **J**_**g**_ and **a** are evaluated at **B**_tot_ = 0. We can identify **χ** as the magnetizability tensor, and call  as the mixed anapole susceptibility tensor, and  as the anapole susceptibility tensor.

When the Hellmann–Feynman theorem is applicable, the expectation value quantities can be equated with energy derivatives
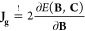
11
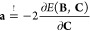
12However, when LAOs are used, the basis set depends on the parameters **B** and **C** leading to a discrepancy between the expectation values and the energy derivatives, in general, except in the complete basis set limit.

Second-order susceptibilities may be defined as follows
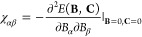
13
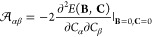
14
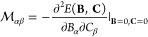
15One can also introduce the closely related, but inequivalent, quantities
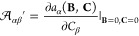
16
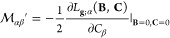
17
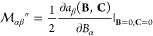
18Again, in the basis set limit, equivalence is restored, i.e.,  and . Note that the multiplicative factors in eqs 10, 15, 17, and 18 have been corrected from those reported in earlier publications^23,25^ to be self-consistent with the other definitions. This implies that the  values reported in these publications should be halved. However, this has no implication on the conclusions of the two papers.

## 3 Results and Discussion

Our test set contains 36 molecules (HF, CO, N_2_, H_2_O, HCN, HOF, LiH, NH_3_, H_2_CO, CH_4_, C_2_H_4_, AlF, CH_3_F, C_3_H_4_, FCCH, FCN, H_2_S, HCP, HFCO, H_2_C_2_O, LiF, N_2_O, OCS, H_4_C_2_O, PN, SO_2_, OF_2_, H_2_, H_2_O_2_, BH, CH^+^, AlH, BeH^–^, SiH^+^, C_4_H_4_, FNO) and subsumes the test set of diamagnetic molecules in Tellgren et al.^12^ and the closed-shell paramagnetic molecules in the test set of Reimann et al.^36^ Geometries for the molecules are as reported in earlier publications^12,23,36^ and are also provided in the Supporting Information.

All calculations were performed using the London program.^20,22^ The density functional calculations used the previously reported implementations.^12,35^ The coupled-cluster calculations were performed using the previously reported implementation^69^ and exploratory calculations (results not included) also used a new functionality.^70^ The symmetric finite difference formula for numerical second derivatives of the energy was employed to compute the anapole susceptibilities  and . Step sizes of ϵ = 0.01 au for **B** and ϵ′ = 0.005 au for **C** were used. ϵ′ was chosen to be smaller as the effect of **C** on the local magnetic field is scaled by the interatomic distances in the molecule. The reference point, **h**, for **C** was placed at the center of charge of the molecules in all cases. The error in the energy is quadratic in the step size within the limits to which the energy is converged while the error in the analytically computed moments (first derivative of energy) is linear. All numerical results presented in this article are given in SI-based atomic units—see the earlier work for the conversion factors to SI units.^23^

The uncontracted aug-cc-pCVTZ basis set has been employed for all of the computations. The name of the basis set is prefixed with “L” to denote the use of London atomic orbitals and “u” to indicate that the basis sets are uncontracted—Luaug-cc-pCVTZ.

### 3.1 Current-Dependence and Meta-GGAs

The meta-GGA functional form allows a dependence on the kinetic energy density. In the absence of a magnetic field, the everywhere positive, canonical kinetic energy τ_can_ = 1/2 ∑_*k*_|∇ϕ_*k*_|^2^, with summation over occupied orbitals ϕ_*k*_, is the natural choice. In the presence of a magnetic field, τ_can_ is gauge dependent and cannot be used. An obvious solution is to use the gauge independent, physical kinetic energy density τ_phys_ = 1/2 ∑_*k*_|(−*i*∇ + **A**_tot_)ϕ_*k*_|^2^ instead. This choice has been suggested by Maximoff and Scuseria.^71^ An alternative, with some theoretical aspects and numerical results in its favor,^36,72−74^ is to instead use Dobson’s gauge-invariant kinetic energy density
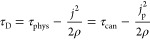
19where **j**_p_ = Im ∑_*k*_ ϕ_k_^*^∇ϕ_*k*_ is the paramagnetic current density and **j** = Re ∑_*k*_ ϕ_k_^*^(−*i*∇ + **A**_tot_)ϕ_*k*_ is the physical current density.

A previous work^36^ used a prefix “a” to denote meta-GGA functionals using the physical τ_phys_ (e.g., aTPSS) and a prefix “c” to denote functionals using Dobson’s τ_D_ (e.g., cTPSS). In the present work, we only consider the latter type, specifically cTPSS and cM06-L.

### 3.2 Classification

Different response tensors in general have different physical dimensions and units, although this fact is somewhat obscured when working with atomic units. To account for this, we fix a length , and define an auxiliary quantity , and auxiliary response tensors  and . In atomic units, the numerical values of the quantities  and  remain unchanged by this transformation of response tensors to shared units. Next, we construct a 6 × 6 matrix of the form
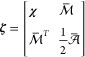
20which allows us to re-express the second-order energy in eq 10 as
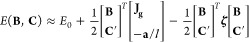
21The tensor **ζ** is symmetric and has real eigenvalues, which we denote by **α**_**ζ**_ = (α_**ζ**;1_, ..., α_**ζ**;6_). *If one or more eigenvalues are positive, i.e., energy decreases with any component of* (**B**, **C**), *we classify the system broadly as paramagnetic. Otherwise, if all eigenvalues are negative, we classify the system as diamagnetic.* Diagonalization of the submatrices gives us further details of this behavior such as separate response to only **B** or **C**. Additionally, the trace of **ζ** gives us a single number for the overall response to a generally nonuniform field. We also obtain an average eigenvalue as
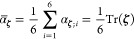
22which is also the orientational average over all possible molecular orientations (with **B**, **C** fixed). This procedure may be extended to increasingly nonuniform magnetic fields, such as those with curvatures and beyond. In this article, the magnetic field has a constant gradient and is of the form shown in eq 1.

**Figure 1:**
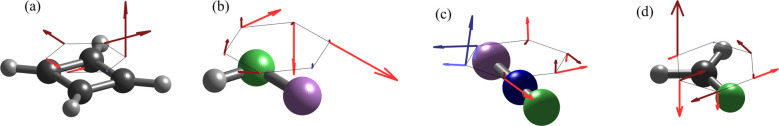
Visualization of six-dimensional eigenvectors (**B**, **C**′) as pairs of three-dimensional vectors for (a) C_4_H_4_, (b) HOF, (c) FNO, and (d) CH_2_O. The length is scaled based on the eigenvalue and red and blue are used for negative (diamagnetic) and positive (paramagnetic) components, respectively. The **C**′ component is indicated with a darker color and different vector pairs start from different vertices on a hexagon.

The different eigenvectors of **ζ** are visualized for four molecules in Figure 1. CCSD level tensors were used for HOF and FNO and MP2 level tensors for C_4_H_4_ and CH_2_O. Each six-dimensional eigenvector (**B**, **C**′) is represented as a pair of three-dimensional vectors, displaced along the vertices of a hexagon to make clear which arrows form pairs. The length of each arrow is proportional to the eigenvalue, though the proportionality constant varies between molecules, and the sign is indicated by red (negative) and blue (positive) colors. In the plot, the arrows are sorted so that the magnitude increases clockwise around the hexagon. Note also that the eigenvectors and the molecular geometry are expressed in the same coordinate system, such that the angle between eigenvectors and bond axes is independent of the choice coordinate system and orientation of the molecule. If the mixed anapole susceptibility tensor vanishes, the eigenvalue problem does not couple the **B** and **C**′. This happens for cyclobutadiene, for which each eigenvector is of the form (**B**, **0**) or (**0**, **C**′). On the other hand, for FNO and CH_2_O, there is substantial coupling and some eigenvectors have **B** and **C**′ components of similar magnitude. Of the molecules shown, C_4_H_4_, HOF, and CH_2_O each have a single paramagnetic eigenvector with a magnitude that is small compared to the diamagnetic eigenvalues—in the plot the corresponding arrows are barely visible. By contrast, FNO has two paramagnetic eigenvalues of large magnitude.

**Figure 2:**
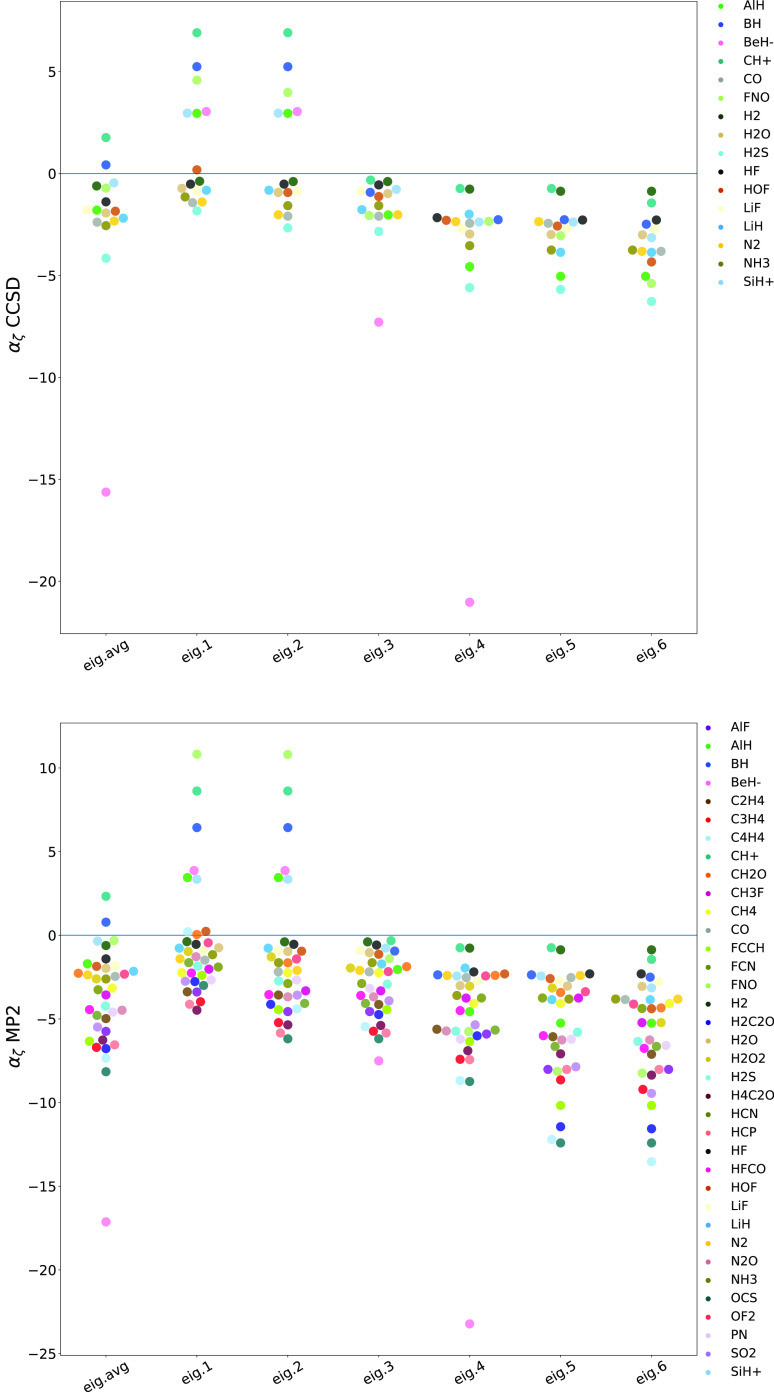
Eigenvalues of ζ in the decreasing order computed with CCSD (top panel) and MP2 (bottom panel) in the Luaug-cc-pCVTZ basis set. eig avg is α̅_ζ_ = 1/6 Tr(ζ). Eigenvalues no. 5 and 6 for BeH^–^ lie beyond the range of the plot: eig5 = eig6 = −71.541 (top panel) and −79.810 (bottom panel).

In Figure 2, we have plotted the eigenvalues, α_ζ_, of the super-tensor, **ζ** in decreasing order. We have presented the values obtained with the most accurate methods we have studied in this article, viz. CCSD and MP2. However, the classification is robust and all methods studied by us including Hartree–Fock (HF) and DFT show the same qualitative classification (except for FNO). The geometry, energy, and properties of FNO are all extremely sensitive to correlation and need at least CCSD(T) level computations for reasonable accuracy.^75^ The values of the susceptibilities cannot be determined with any reasonable accuracy with our set of methods. Moreover, the computations do not converge with LDA and cM06-L functionals. We have thus left this molecule out of the error statistics presented in Section 3. All conventionally paramagnetic molecules show at least one positive eigenvalue. In addition, FNO and HOF show positive eigenvalues arising from a paramagnetic response to some component of **C**. Thus, according to our criterion, the following molecules from our test set are paramagnetic: AlH, BH, BeH^–^, C_4_H_4_, CH^+^, CH_2_O, SiH^+^, HOF, and FNO. The nature of this net paramagnetic behavior is summarized in Table 1.

**Table 1:** Selected Molecules Which Are Proposed to Be Classified by Us as Paramagnetic^a^

	**B**		**C**		(**B**, **C**)	
molecule	α̅_χ_	α_χ;max_			α̅_ζ_	α_ζ;max_
AlH	+	+	–	–	–	+
BH	+	+	–	–	+	+
BeH^–^	+	+	–	–	–	+
C_4_H_4_	+	+	–	–	–	+
CH^+^	+	+	–	–	+	+
CH_2_O	–	+	–	–	–	+
FNO	–	–	+	+	–/+^b^	+
HOF	–	–	–	+	–	+
SiH^+^	+	+	–	–	–	+

a For response tensors related to **B**, **C**, and jointly to (**B**, **C**), we show the sign of the average and maximum eigenvalue.

b The molecule FNO is extremely challenging for correlated theories, leading to different conclusions from different methods with respect to the net response to (**B**, **C**): diamagnetic (−) from CCSD and MP2; paramagnetic (+) from HF, KT3, and cTPSS; and not converged for LDA and cM06-L.

We note that, due to the Cauchy interlace theorem, adding dimensions will increase the maximum eigenvalue and decrease the minimum eigenvalue. Hence, with α_**ζ**;max_ = max_1≤*i*≤6_α_**ζ**;*i*_, α_**χ**;max_ = max_1≤*i*≤3_α_**χ**;*i*_, and , we have
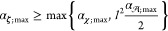
23With a similar notation for the minimum eigenvalues, we get an inequality in the reverse direction. When , usually by reason of symmetry, equality is achieved such as for C_2_H_4_, C_4_H_4_, CH_4_, H_2_, and N_2_. Equality is also achieved for the largest eigenvalue of **ζ** in CH_2_O, CO, and HCP. The smallest eigenvalue also almost saturates in these cases. This may indicate that there is a limit to how large the orbital paramagnetic and/or diamagnetic response can be within the limits of the basis set and molecular symmetry. While the non-zero components of  are small (∼10^–2^ au) for CO and HCP, this is not the case for CH_2_O (,  au).

Most molecules studied are highly diamagnetic with respect to **C** but we must remember that here we are only studying the orbital response. The spin symmetry breaking caused by **C** will activate the spin-Zeeman term in the Hamiltonian leading to an overwhelmingly paramagnetic response to **C**.^25^ The interplay of spin and orbital effects of nonuniform magnetic fields is discussed in an earlier publication.^25^ BeH^–^ is particularly strongly diamagnetic to **C**. The DFT computation with the cM06-L functional does not converge for BeH^–^. FNO, on the other hand, is strongly paramagnetic with respect to **C** according to computations with all of the methods in our study.

### 3.3 Performance of Density Functional Approximations for Magnetic Susceptibilities

In this section, we discuss the relative performance of various wavefunction and density functional methods in computing anapole magnetizabilities,  and . We compare this performance with their accuracy in describing the magnetizability, χ—the most well-studied among the magnetic response quantities.

To quantify errors in the response tensors relative to a reference method we rely on the Frobenius norm
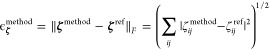
24and similarly for **χ**, , and . For the isotropic average, we use a similar notation to mean ϵ_α̅_**ζ**__^method^ = |α̅_**ζ**_^method^ – α̅_**ζ**_^ref^|.

We present the error bars for the various methods as box and whisker plots where the median error is indicated by the horizontal line in the middle of the box and the top and bottom ends of the box indicate the third quartile and first quartile, respectively. The length of the box is thus the interquartile range. The top and bottom ends of the whiskers indicate the maximum and minimum errors considered in the estimation of the quartiles. Points beyond the whiskers are not considered in the statistics and are regarded as outliers. We superimpose swarmplots on the box and whisker plots to display the underlying data.

**Figure 3:**
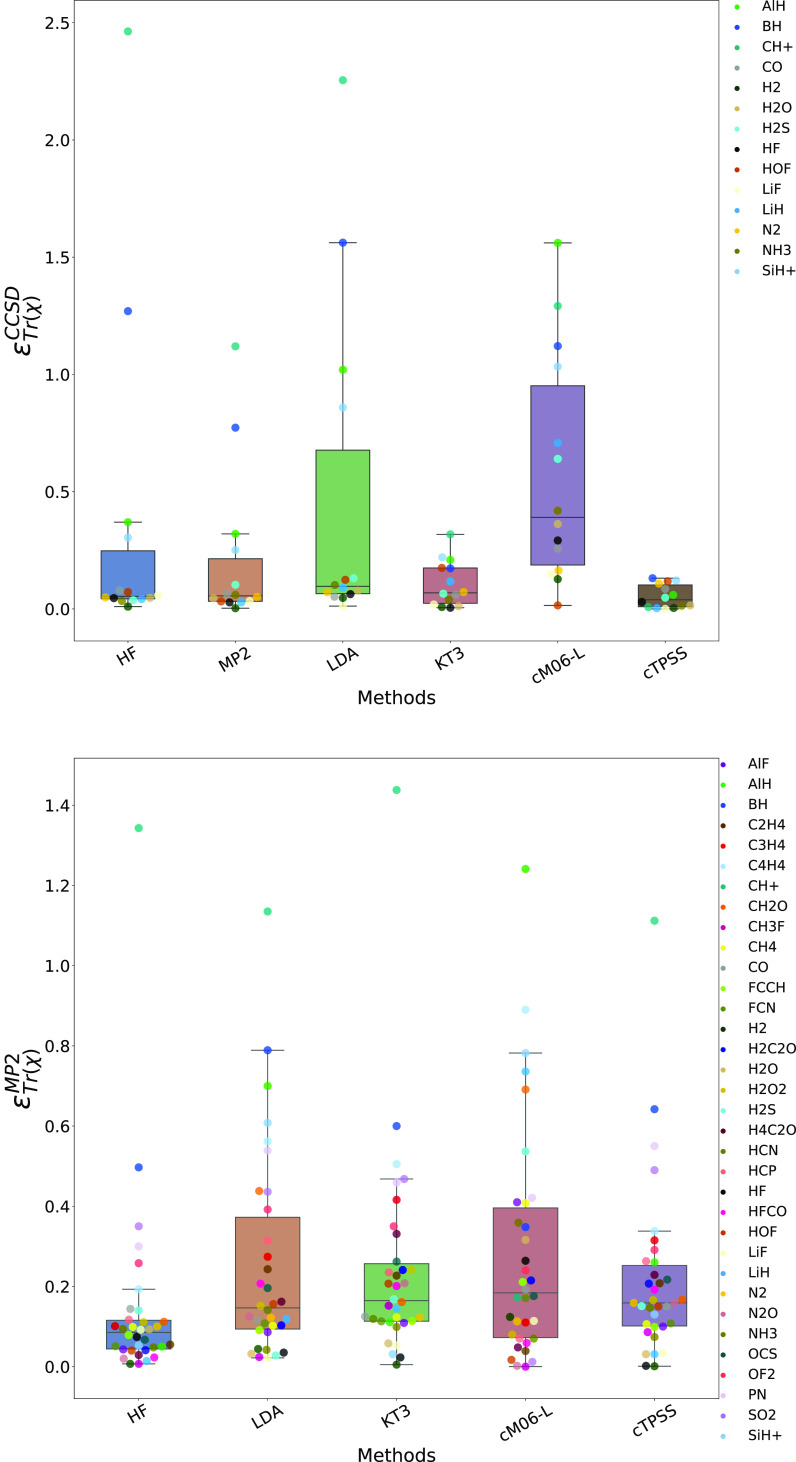
Errors in the isotropic magnetizability computed by various methods in the aug-cc-pCVTZ basis set relative to CCSD (top panel) and MP2 (bottom panel).

Values of isotropic magnetizability computed with Hartree–Fock theory are often reasonable in the absence of low-lying excited states with correlation contributions of the order of 1–3%.^76^ However, DFT approximations, which are reasonably good for correlation energy and electric properties, such as BLYP or B3LYP, are mostly inaccurate for magnetic properties, often being worse than Hartree–Fock theory. This has prompted the development of exchange–correlation functionals tailored to magnetic properties such as the KT3 functional of Keal and Tozer.^13^ cTPSS has also been seen to be remarkably accurate for the same.^12^ Computational studies have indicated that the effect of electron correlation on the isotropic magnetizability (Tr(**χ**)/3)^76^ is often an order of magnitude lower than that on the anisotropic magnetizability.^77^ The isotropic magnetizability is also less sensitive to the basis set size. These conclusions are borne out by our results, as shown in Figure 3. We have not studied BLYP and B3LYP as they are known to perform poorly for conventional magnetic properties.

While the accuracy of various DFT functionals for magnetizability has been well-studied, it remains to be seen if the same conclusions can be reached for more exotic properties such as anapole susceptibilities. In particular, we wish to explore if the KT3 and cTPSS functionals continue to perform well for  and .

**Figure 4:**
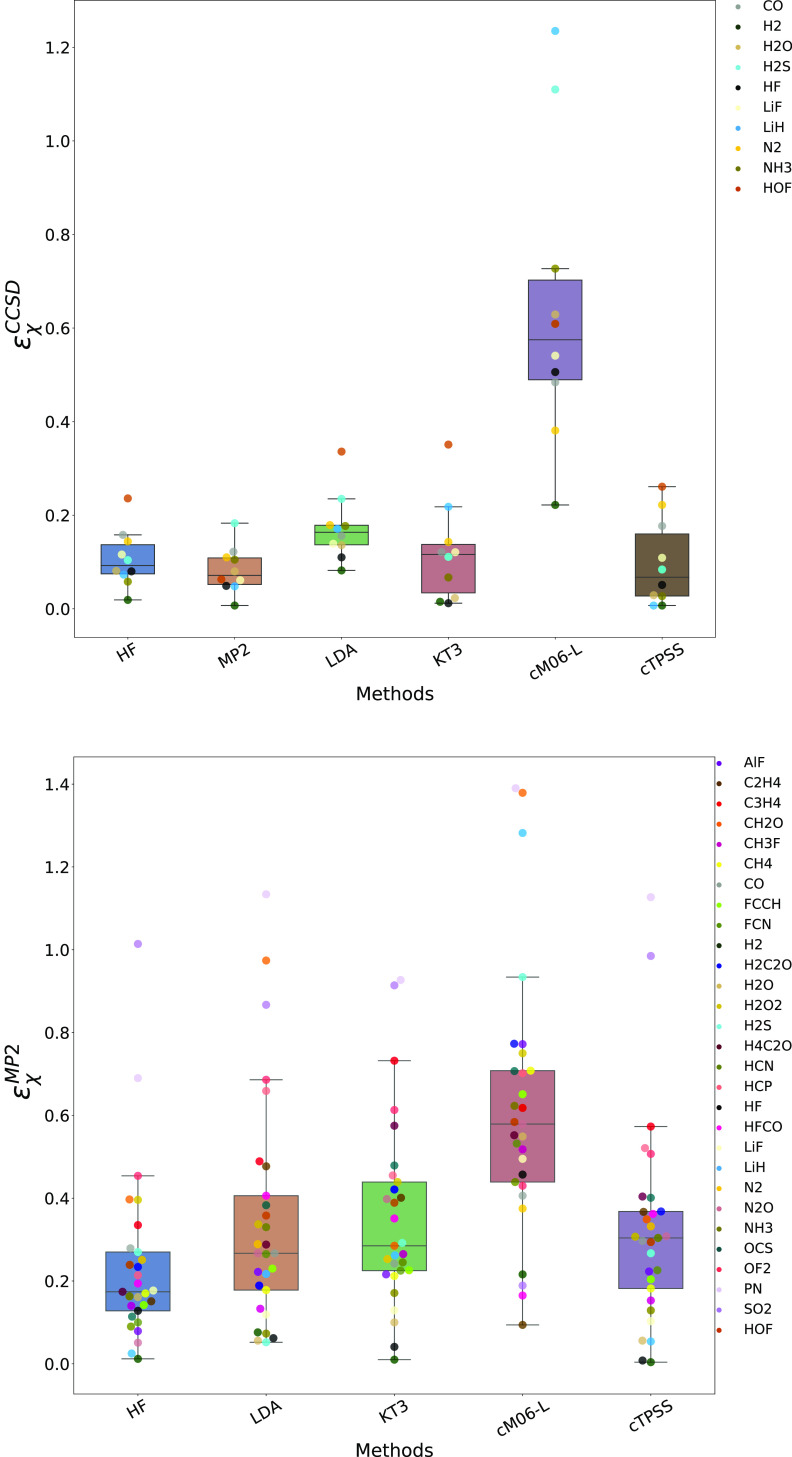
Errors in magnetizability tensor, χ, of the conventionally diamagnetic molecules (negative α̅_χ_) in our test set computed by various methods in the Luaug-cc-pCVTZ basis set relative to CCSD (top panel) and MP2 (bottom panel).

**Figure 5:**
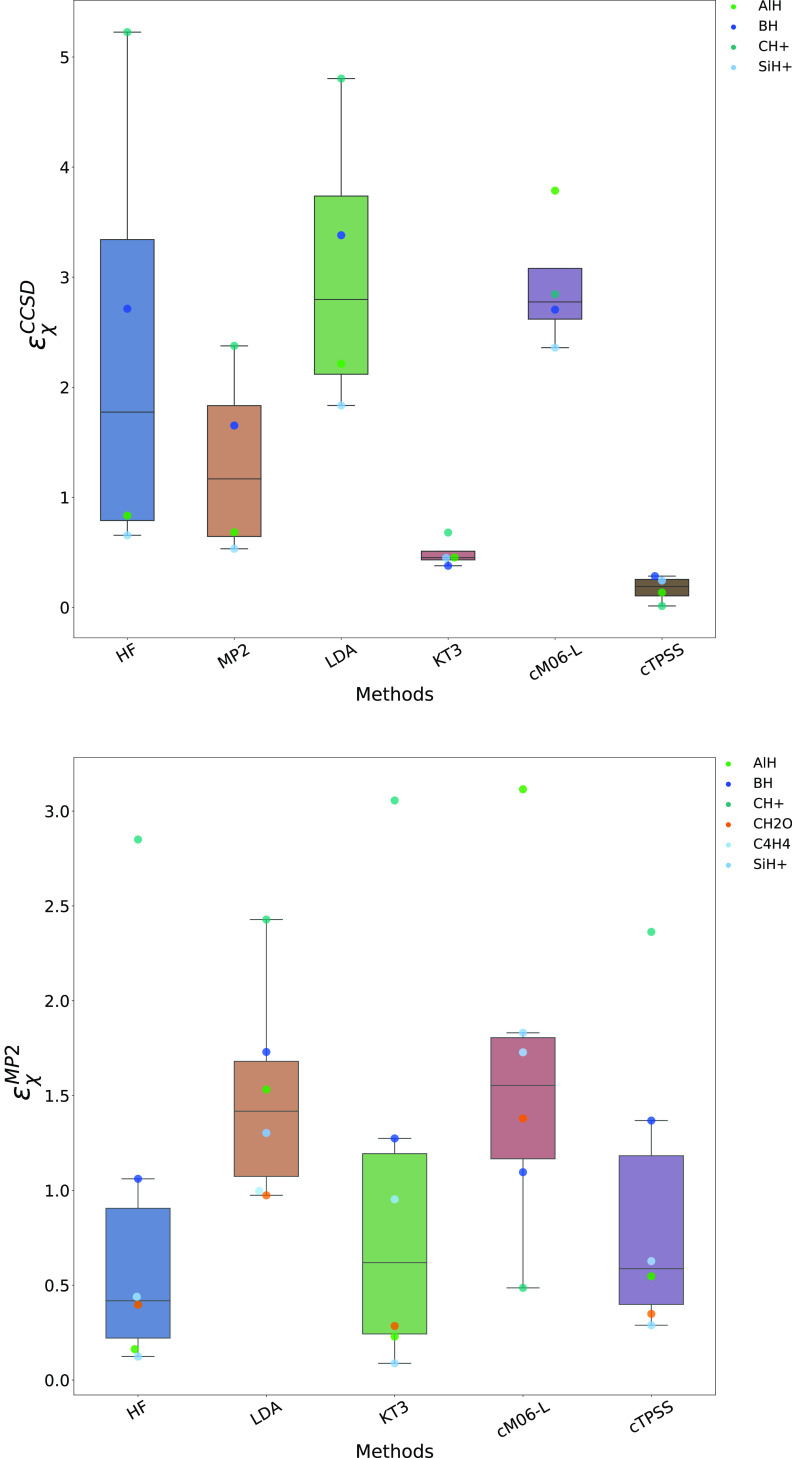
Errors in the magnetizability tensor, χ, of the conventionally paramagnetic molecules (positive α̅_χ_) in our test set computed by various methods in the Luaug-cc-pCVTZ basis set relative to CCSD (top panel) and MP2 (bottom panel).

In the top panel of Figure 4, we can see that except the cM06-L functional all of the methods considered here perform reasonably for the conventionally diamagnetic molecules. The cTPSS functional and MP2 show similar accuracy with HF and KT3 also doing quite well. The bottom panel in Figure 4 samples a larger test set using MP2 as the benchmark. Among the density functionals LDA, KT3, and cTPSS perform similarly. It is interesting to note that the outlier for many of the methods in the top panel is HOF, which would be classified as paramagnetic by our proposed scheme. The trends for the paramagnetic molecules plotted separately in Figure 5 are much more surprising. All of the errors are much higher than for the diamagnetic molecules indicating how much more difficult it is to describe the paramagnetic response. MP2 no longer performs as well and is easily surpassed in accuracy by both KT3 and cTPSS. A similar conclusion was reached by Reimann et al.^36^ where even CCSD performed worse than cTPSS relative to CCSD(T) for the paramagnetic molecules. The bottom panel in Figure 5 is thus much less reliable as a measure of errors in density functionals. Between KT3 and cTPSS, cTPSS is somewhat more accurate for the paramagnetic systems studied by us.

**Figure 6:**
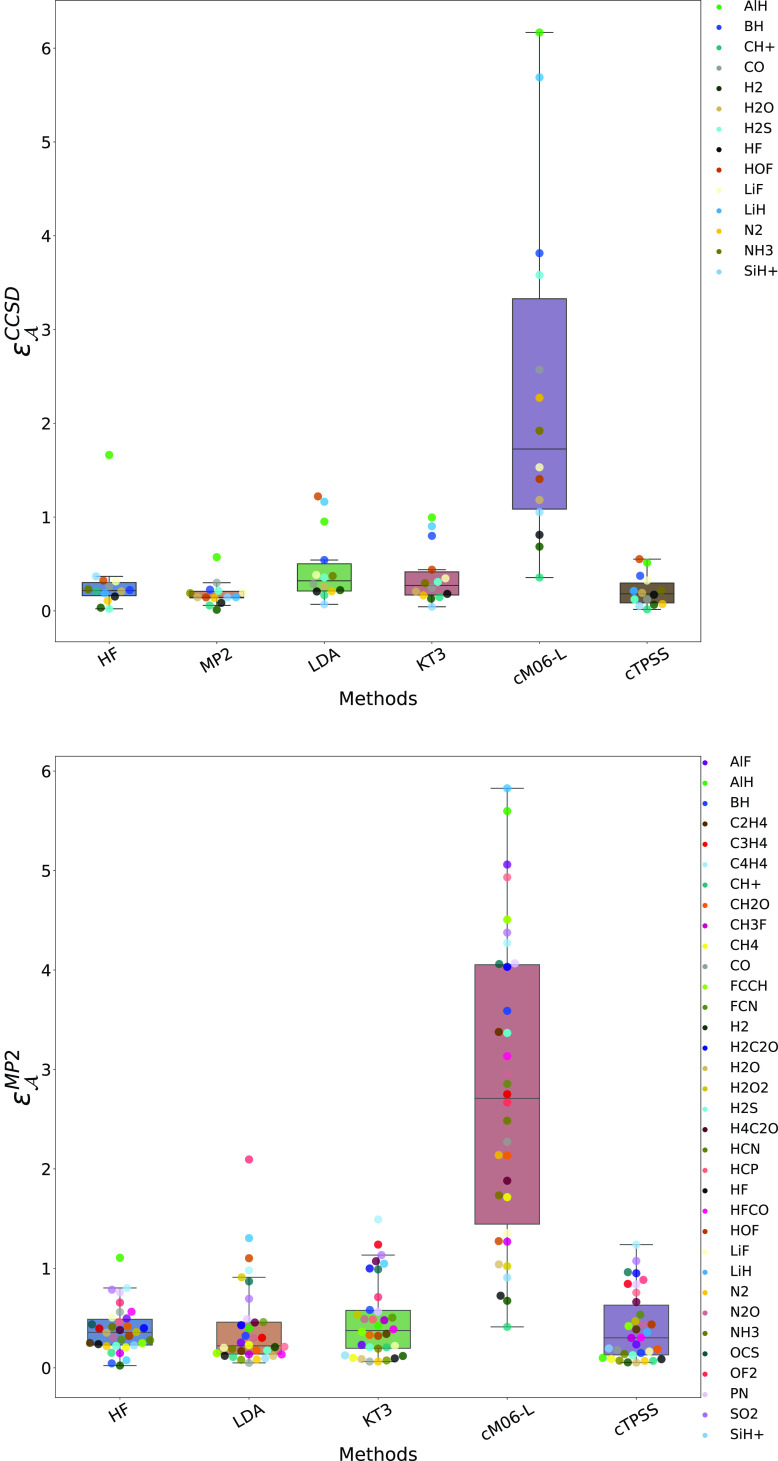
Errors in anapole susceptibility, , computed by various methods in the Luaug-cc-pCVTZ basis set relative to CCSD (top panel) and MP2 (bottom panel).

The top panel of Figure 6 shows a reasonable description of  by most methods (except cM06-L) against CCSD. MP2 shows the highest accuracy with cTPSS following close behind. The bottom panel of Figure 6 also follows the same trends with MP2 as the reference. Here too, the paramagnetic molecules show a larger error than the diamagnetic ones with HOF behaving as the conventionally paramagnetic molecules.

**Figure 7:**
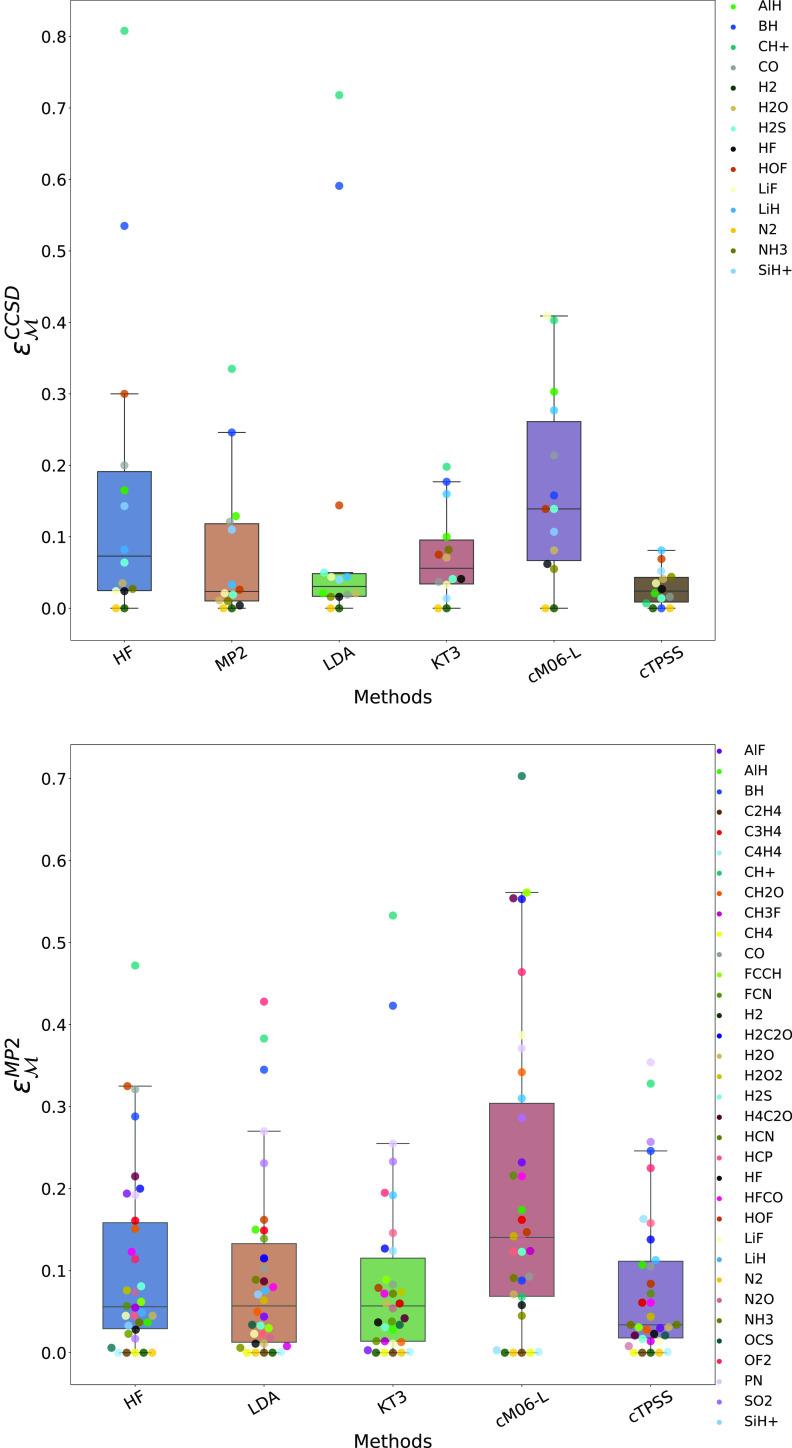
Errors in mixed anapole susceptibility, , computed by various methods in the Luaug-cc-pCVTZ basis set relative to CCSD (top panel) and MP2 (bottom panel).

Due to the smaller numerical values in the mixed anapole susceptibility tensor, , the errors appear to be smaller in Figure 7. cM06-L is no longer as bad although it is still the worst among the methods studied.

**Figure 8:**
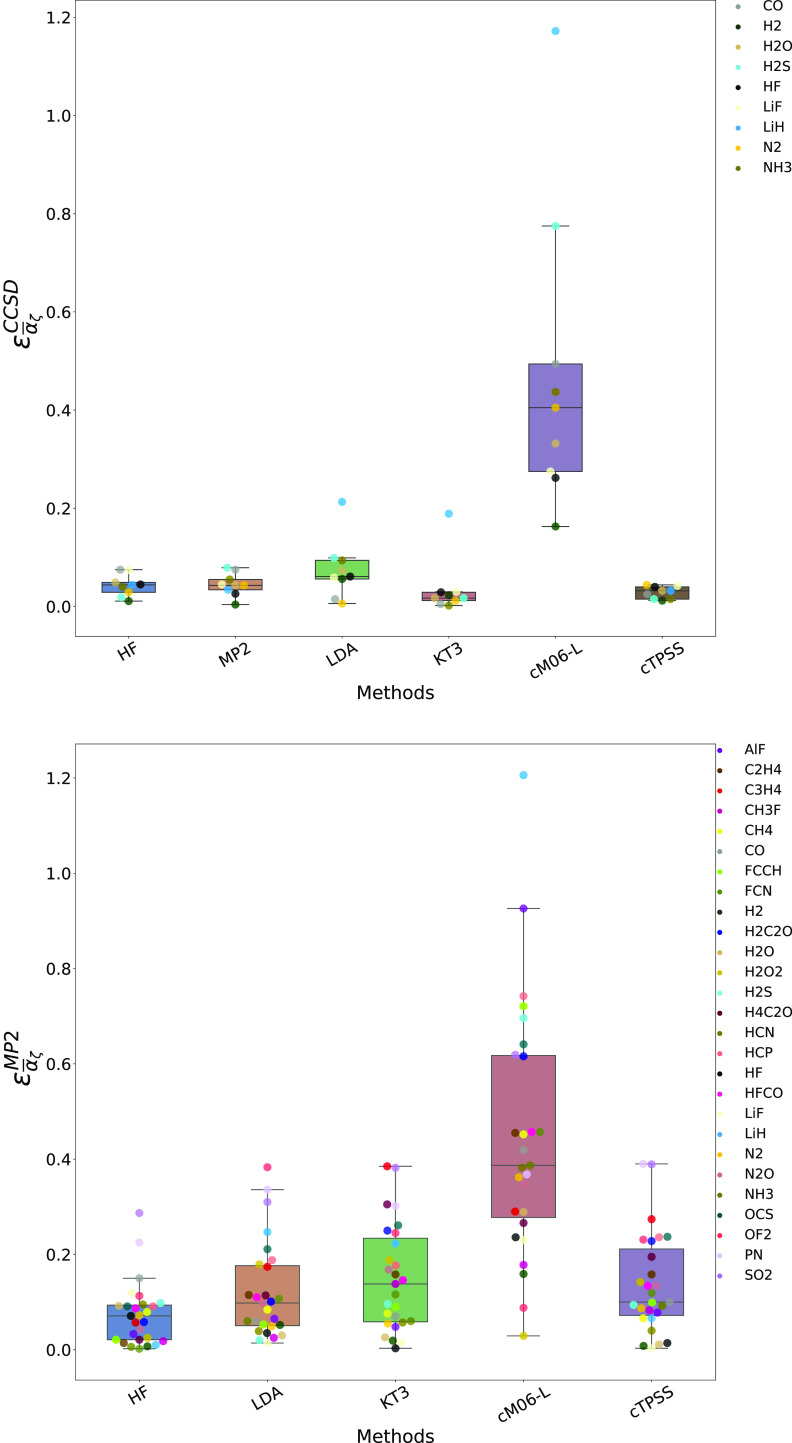
Errors in the average eigenvalue of the super-tensor, ζ, computed by various methods in the Luaug-cc-pCVTZ basis set relative to CCSD (top panel) and MP2 (bottom panel) for diamagnetic molecules as classified by us.

**Figure 9:**
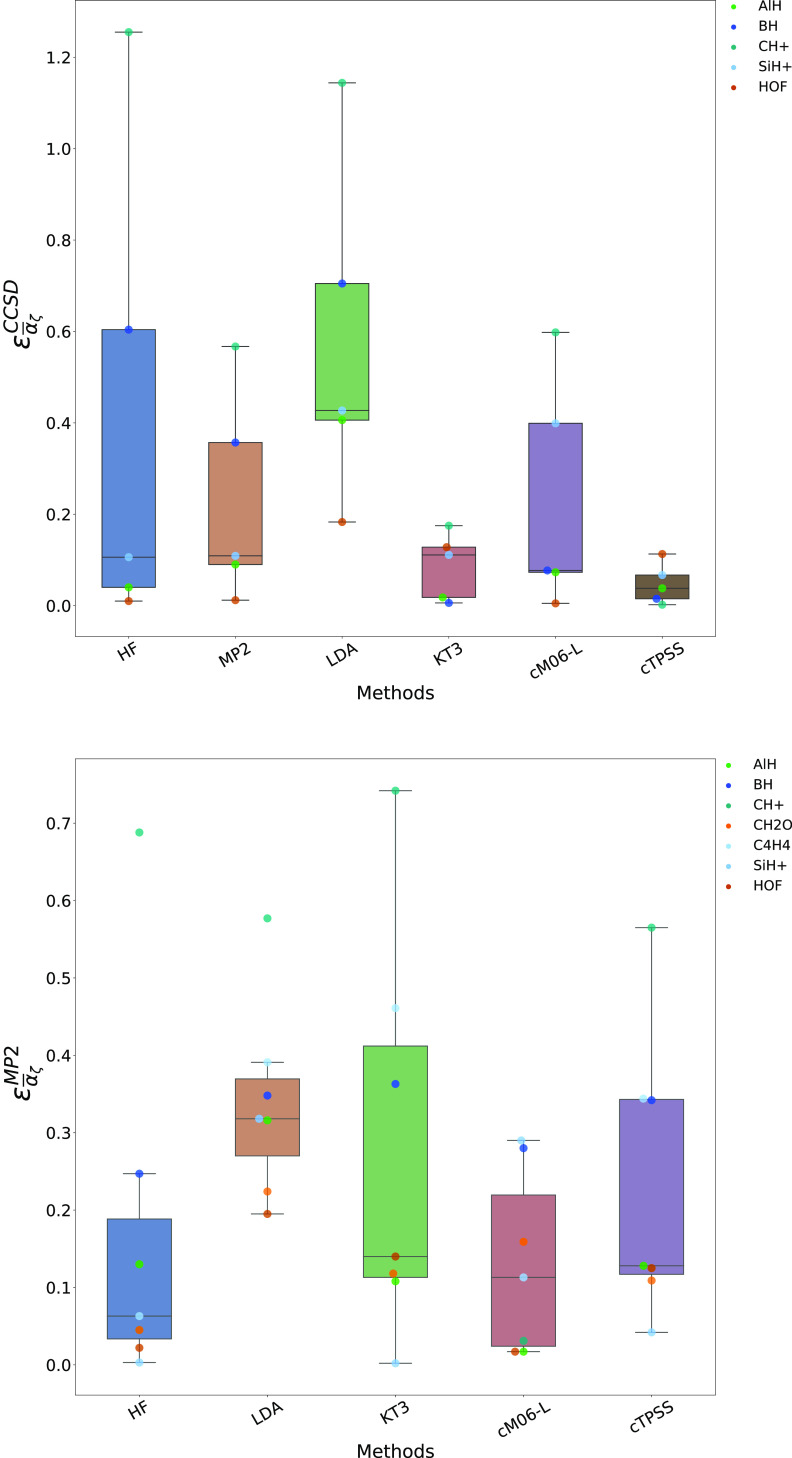
Errors in the average eigenvalue of the super-tensor, ζ, computed by various methods in the Luaug-cc-pCVTZ basis set relative to CCSD (top panel) and MP2 (bottom panel) for paramagnetic molecules as classified by us.

Finally, we present the errors in the quantity, α̅_ζ_—the average eigenvalue of our super-tensor, ζ in Figures 8 and 9. The molecules have been classified according to the criterion in Section 3.2. This may be considered as a condensed representation of all of the errors presented in Figures 3–7 allowing for the possibility of some error cancellation. The top panel indicates a comparable performance of MP2, KT3, and cTPSS in comparison with CCSD. The larger test set in bottom panel also fits with a similar performance. Molecules classified as paramagnetic by us again show the largest errors.

Our findings are summarized in Table 2.

**Table 2:** Methods Studied by Us Relative to CCSD in the Increasing Order of Median Errors

property	class	error
Tr(χ)	dia	cTPSS < MP2 < HF ≈ KT3 < LDA ≪ cM06-L
Tr(χ)	para	cTPSS ≈ KT3 < MP2 < HF < LDA ≈ cM06-L
χ	dia	cTPSS < MP2 ≈ HF < KT3 < LDA ≪ cM06-L
χ	para	cTPSS < KT3 < MP2 < HF ≪ LDA ≈ cM06-L
	dia	MP2 ≈ cTPSS < HF < KT3 < LDA ≪ cM06-L
	para	MP2 < HF ≈ cTPSS < KT3 < LDA ≪ cM06-L
	dia	MP2 < LDA < cTPSS < HF < KT3 < cM06-L
	para	cTPSS < KT3 < MP2 ≈ LDA < HF ≈ cM06-L
α̅_ζ_	dia	cTPSS ≈ KT3 < MP2 ≈ HF < LDA ≪ cM06-L
α̅_ζ_	para	cTPSS ≈ MP2 ≈ KT3 < HF < LDA < cM06-L

### 3.4 Performance of Density Functional Approximations for Nonperturbative Effects

As we have seen in the previous subsection, molecules which show paramagnetic behavior, with respect to a component of **B** or **C**, are particularly challenging for all of the theories. In this subsection, we further explore two molecules—BH as an example of paramagnetic behavior with respect to a component of **B** and HOF as the newly discovered example of paramagnetic behavior with respect to a component of **C**.

**Figure 10:**
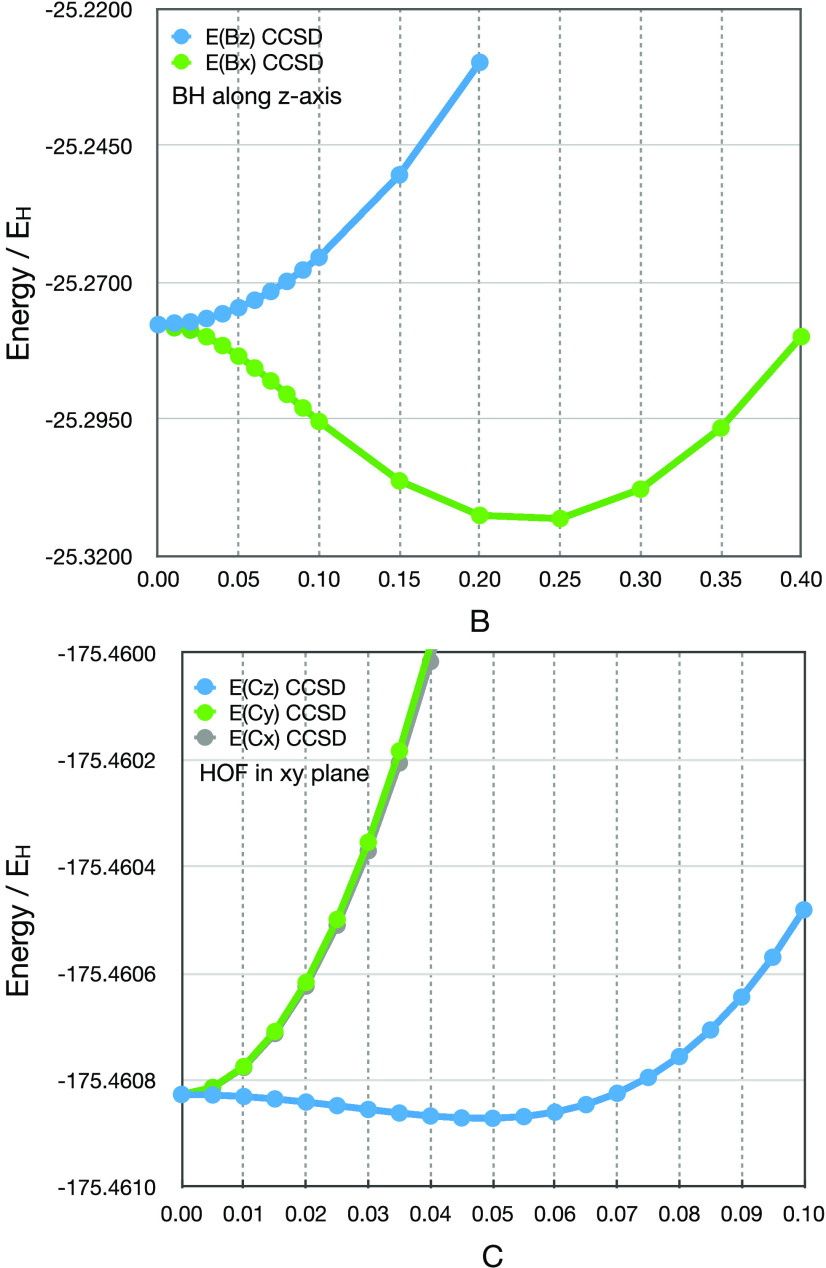
Variation of the energy of BH with a uniform field, **B** (top panel) and HOF with the curl of the field, **C** (bottom panel) showing an initial paramagnetic orbital response and eventually the quadratic Zeeman effect in both cases.

In the top panel of Figure 10, the paramagnetic behavior of BH with respect to a field (*B*_*x*_) perpendicular to the bond axis (*z*) is evident up to a critical field strength of about *B*_*x*_ = 0.22 au after which the quadratic term takes over. An analogous behavior is observed in HOF (bottom panel of Figure 10) with increase in the component of **C** (*C*_*z*_) perpendicular to the molecular plane (*xy*). The turning point can be read off as *C*_*z*_ = 0.048 au. The depth of the minimum is, however, only 10^–5^*E*_H_ for HOF against a depth of 10^–2^*E*_H_ for BH.

**Figure 11:**
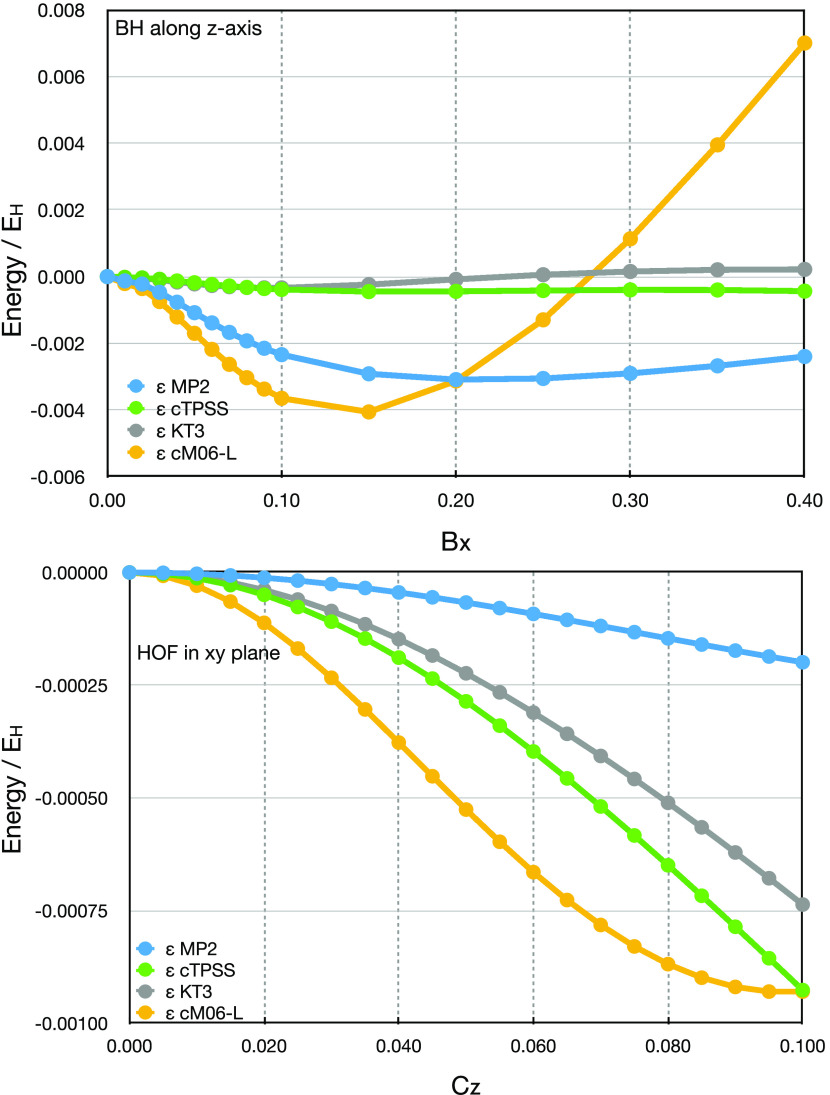
Errors in energy, ε relative to CCSD for BH with a uniform field, *B*_*x*_ (top panel) and HOF with the curl of the field, and *C*_*z*_ (bottom panel). All energies have been shifted by the corresponding zero-field values such that all plots start at ε = 0.

We have tried to assess the capacity of various theories to describe the changes in the electronic structure arising from the application of increasing **B** and **C**. The CCSD method has been chosen as the reference and energies are computed with MP2 and a few selected density functional approximations. Since the zero-field energies themselves differ considerably, we have subtracted the zero-field energy computed with each method from all other data points thereby shifting all plots to a common starting point of zero. The energy difference between these shifted data points of various methods and CCSD is then plotted in Figure 11. For BH (top panel), cTPSS and KT3 work best with nearly parallel error curves. MP2 is surprisingly worse and the cM06-L functional gives a highly non-parallel error plot. For HOF (bottom panel), the error values themselves are an order of magnitude smaller than for BH with MP2 showing the best performance. KT3 and cTPSS follow the same trend of errors increasing with increasing *C*_*z*_ as MP2. No flattening is observed even when HOF starts showing diamagnetic behavior after the turning point of *C*_*z*_ = 0.048 au unlike the plots for BH. The cM06-L functional yields a very non-parallel error curve in this case too. Although not shown here, the corresponding plots for BH vs *C*_*i*_, *i* = *x*, *y*, *z*, also show increasing errors with increasing *C*_*i*_.

## 4 Summary and Conclusions

In this article, we have suggested a new classification of magnetic behavior of molecules based on their response to a generally nonuniform field. We have demonstrated that paramagnetic behavior can arise in a molecule due to inhomogeneities in the field even when its response to a uniform field is diamagnetic as is the case for FNO and HOF. We have concluded that the susceptibilities of molecules—**χ**,  and —thus classified as paramagnetic are more difficult to describe. Assuming that CCSD gives accurate results, KT3 and cTPSS are found to show the best performance among the DFT approximations with cTPSS being marginally better than KT3. The interquartile range for both functionals are narrow across all of the properties studied by us. cTPSS and KT3 also perform quite well for the more challenging paramagnetic molecules, even better than MP2 relative to CCSD. cM06-L is particularly bad for magnetic properties performing even worse than LDA. These conclusions are also found to hold in the strong-field regime as verified for some typical challenging molecules. Hartree–Fock is surprisingly reliable for diamagnetic molecules with a more or less constant error across all magnetic properties. The paramagnetic molecules are far more sensitive to correlation. The eigenvalues of **ζ**, or even its higher dimensional analogues with additional parameters beyond **B** and **C**, can serve as a concise measure for comparing the accuracy of various theories in describing general magnetic properties.

## Supporting Information Available

Molecular geometries and data corresponding to the plots in this article have been tabulated (PDF)
