# Supplementary material for: Benchmarking Density Functional Approximations for Diamagnetic and Paramagnetic Molecules in Nonuniform Magnetic Fields
Source: J Chem Theory Comput. 2021 Feb 12;17(3):1480–96. doi: 10.1021/acs.jctc.0c01222 (PMC7948255; doi:10.1021/acs.jctc.0c01222)
Supplement: Supplementary file 1 — ct0c01222_si_001.pdf [file ct0c01222_si_001.pdf]

Supplementary Information: Correlated computations of magnetic anapole susceptibilities of molecules and their classification on the basis of response to generally non-uniform magnetic fields

Sangita Sen* and Erik I. Tellgren*

Hylleraas Centre for Quantum Molecular Sciences, Department of Chemistry, University of Oslo, P.O. Box 1033 Blindern, N-0315 Oslo, Norway

E-mail: sangita.sen@kjemi.uio.no; erik.tellgren@kjemi.uio.no

1 Geometries

The Cartesian coordinates in bohr are listed below for the molecules included in this study.

AlF

Al	0.000000	0.000000	-1.308405
F	0.000000	0.000000	1.856777

AlH

Al	0.000000	0.000000	0.000000
H	0.000000	0.000000	2.079820

HFCO

O	-2.087492	0.692863	0.000000
C	-0.377439	-0.739889	0.000000
F	2.038239	0.001906	0.000000
H	-0.466335	-2.791172	0.000000

HOF

BH

B 0.000000 0.000000 0.000000

H 0.000000 0.000000 2.334190

BeH-

Be 0.000000 0.000000 0.000000

H 0.000000 0.000000 2.682830

C2H4

C 0.000000 1.259465 0.000000

C 0.000000 -1.259465 0.000000

H 1.740064 2.321626 0.000000

H -1.740064 2.321626 0.000000

H 1.740064 -2.321626 0.000000

H -1.740064 -2.321626 0.000000

O -1.418692 0.109003 0.000000

H -1.731365 -1.689574 0.000000

F 1.289927 0.003159 0.000000

LiF

Li 0.000000 0.000000 -2.173222

F 0.000000 0.000000 0.803950

LiH

H 0.000000 0.000000 2.637010

Li 0.000000 0.000000 -0.378734

N2

N 0.000000 0.000000 1.039909

N 0.000000 0.000000 -1.039909

C3H4

C 0.000000 0.000000 1.665529

C 0.000000 1.224758 -0.906882

C 0.000000 -1.224758 -0.906882

H 0.000000 2.975838 -1.918779

H 0.000000 -2.975838 -1.918779

N2O

N 0.000000 0.000000 -2.268420

N 0.000000 0.000000 -0.134930

O 0.000000 0.000000 2.104236

H2O

H	1.719445	0.000000	2.777316
H	-1.719445	0.000000	2.777316

C4H4

C	1.475876	1.274620	0.000000
C	-1.475876	1.274620	0.000000
C	1.475876	-1.274620	0.000000
C	-1.475876	-1.274620	0.000000
H	2.912489	2.716258	0.000000
H	-2.912489	2.716258	0.000000
H	2.912489	-2.716258	0.000000
H	-2.912489	-2.716258	0.000000

CH+

C	0.000000	0.000000	0.000000
H	0.000000	0.000000	2.138600

CH2O

O	0.000000	0.000000	-1.141647
C	0.000000	0.000000	1.138301
H	0.000000	1.762395	2.235653
H	0.000000	-1.762395	2.235653

CH3F

O	0.000000	0.000000	-0.136392
H	0.000000	1.423659	0.981343
H	0.000000	-1.423659	0.981343

H2O2

H	0.902593	-1.563337	-1.726149
H	0.902593	1.563337	1.726149
O	0.000000	0.000000	-1.407846
O	0.000000	0.000000	1.407846

H2S

S	0.000000	0.000000	-0.110773
H	0.000000	1.822447	1.640654
H	0.000000	-1.822447	1.640654

H4C2O

O	0.000000	0.000000	1.518240
C	0.000000	1.382495	-0.796276
C	0.000000	-1.382495	-0.796276
H	1.731975	2.379466	-1.212540
H	-1.731975	2.379466	-1.212540
H	1.731975	-2.379466	-1.212540
H	-1.731975	-2.379466	-1.212540

HCN

C	-1.415757	0.000027	0.000000
F	1.188948	0.000010	0.000000
H	-2.089940	-1.939604	0.000000
H	-2.089922	0.969848	1.679773
H	-2.089922	0.969848	-1.679773

CH4

C	0.000000	0.000000	0.000000
H	0.000000	1.675468	1.184736
H	0.000000	-1.675468	1.184736
H	1.675468	0.000000	-1.184734
H	-1.675468	0.000000	-1.184734

CO

C	0.000000	0.000000	1.212532
O	0.000000	0.000000	-0.927517

FCCH

C	0.000000	0.000000	2.592052
C	0.000000	0.000000	0.326613
H	0.000000	0.000000	4.585734
F	0.000000	0.000000	-2.088915

FCN

F	0.000000	0.000000	2.064937
C	0.000000	0.000000	-0.328104
N	0.000000	0.000000	-2.519310

H	0.000000	0.000000	3.065393
C	0.000000	0.000000	1.059657
N	0.000000	0.000000	-1.126448

HCP

H	0.000000	0.000000	4.026662
C	0.000000	0.000000	2.011473
P	0.000000	0.000000	-0.916741

HF

H	0.000000	0.000000	1.644119
F	0.000000	0.000000	-0.087217

NH3

N	0.140247	-0.000044	0.000000
H	-0.596990	1.763051	0.000000
H	-0.597203	-0.881503	1.526834
H	-0.597203	-0.881503	-1.526834

OCS

O	0.000000	0.000000	3.183208
C	0.000000	0.000000	0.993454
S	0.000000	0.000000	-1.971392

OF2

O	0.000000	0.000000	1.162482
F	0.000000	2.082491	-0.489006

FNO

F	2.341900	1.640800	0.000000
N	0.000000	0.000000	0.000000
O	-1.755500	-1.229900	0.000000

H2

H	0.000000	0.000000	0.00000
H	0.000000	0.000000	1.39840

H2C2O

C	0.000000	0.000000	-2.442179
C	0.000000	0.000000	0.038704
O	0.000000	0.000000	2.238037
H	0.000000	1.772139	-3.428402
H	0.000000	-1.772139	-3.428402

F	0.000000	-2.082491	-0.489006

PN

N	0.000000	0.000000	-1.951610
P	0.000000	0.000000	0.889157

SO2

S	0.000000	0.000000	-0.703245
O	0.000000	2.355745	0.692198
O	0.000000	-2.355745	0.692198

SiH+

Si	0.000000	0.000000	0.000000
H	0.000000	0.000000	2.85101

2 Magnetic response tensors

Table 1: χ computed with CCSD in aug-cc-pCVTZ basis

AlH	2.896	0.000	0.000	H2S	-5.499	0.000	0.000
	0.000	2.896	-0.000		0.000	-6.169	-0.000
	0.000	-0.000	-4.565		0.000	-0.000	-5.685
BH	5.122	0.000	0.000	HF	-2.271	-0.000	-0.000
	0.000	5.122	-0.000		-0.000	-2.271	0.000
	0.000	-0.000	-2.485		-0.000	0.000	-2.163
BeH-	2.838	0.000	0.000	HOF	-4.332	0.002	-0.000
	0.000	2.838	-0.000		0.002	-2.188	-0.000
	0.000	-0.000	-7.289		-0.000	-0.000	-2.563
CH+	6.757	0.000	0.000	LiF	-2.361	0.000	-0.000
	0.000	6.757	0.000		0.000	-2.361	-0.000
	0.000	0.000	-1.441		-0.000	-0.000	-2.717
CO	-2.100	-0.000	-0.000	LiH	-1.450	-0.000	-0.000
	-0.000	-2.102	0.000		-0.000	-1.450	0.000
	-0.000	0.000	-3.815		-0.000	0.000	-1.992
FNO	-4.306	-1.554	0.082	N2	-2.020	-0.000	0.000
	-1.554	-2.920	0.116		-0.000	-2.020	0.000
	0.082	0.116	-1.638		0.000	0.000	-3.816
H2	-0.867	0.000	-0.000	NH3	-3.537	-0.000	-0.000
	0.000	-0.867	-0.000		-0.000	-3.748	-0.000
	-0.000	-0.000	-0.763		-0.000	-0.000	-3.748
H2O	-2.993	0.000	0.000	SiH+	2.948	0.000	0.000
	0.000	-2.952	0.000		0.000	2.948	0.000
	0.000	0.000	-2.988		0.000	0.000	-3.142

Table 2: χ computed with MP2 in aug-cc-pCVTZ basis

AlF	-4.719	-0.000	0.000	FCCH	-5.305	-0.000	-0.000	HFCO	-3.591	-0.032	-0.000
	-0.000	-4.719	-0.000		-0.000	-5.305	-0.000		-0.032	-3.597	-0.000
	0.000	-0.000	-5.903		-0.000	-0.000	-6.351		-0.000	-0.000	-4.730
AlH	3.378	0.000	-0.000	FCN	-4.280	-0.000	0.000	HOF	-4.386	0.005	-0.000
	0.000	3.378	0.000		-0.000	-4.280	-0.000		0.005	-2.216	-0.000
	-0.000	0.000	-4.567		0.000	-0.000	-5.665		-0.000	-0.000	-2.577
BH	6.290	-0.000	-0.000	FNO	-5.045	-1.006	-0.000	LiF	-2.384	-0.000	-0.000
	-0.000	6.290	-0.000		-1.006	-4.323	-0.000		-0.000	-2.384	-0.000
	-0.000	-0.000	-2.502		-0.000	-0.000	-3.608		-0.000	-0.000	-2.769
BeH-	3.680	-0.000	-0.000	H2	-0.870	-0.000	-0.000	LiH	-1.424	-0.000	-0.000
	-0.000	3.680	-0.000		-0.000	-0.870	-0.000		-0.000	-1.424	-0.000
	-0.000	-0.000	-7.505		-0.000	-0.000	-0.768		-0.000	-0.000	-1.961
C2H4	-3.570	0.000	0.000	H2C2O	-5.184	-0.000	-0.000	N2	-2.098	-0.000	-0.000
	0.000	-4.136	0.000		-0.000	-5.383	-0.000		-0.000	-2.098	0.000
	0.000	0.000	-5.617		-0.000	-0.000	-6.009		-0.000	0.000	-3.813
C3H4	-8.643	0.000	0.000	H2O	-3.041	-0.000	-0.000	N2O	-3.684	0.000	-0.000
	0.000	-4.118	0.000		-0.000	-2.996	-0.000		0.000	-3.684	0.000
	0.000	0.000	-5.734		-0.000	-0.000	-3.035		-0.000	0.000	-5.720
C4H4	-5.456	-0.000	0.000	H2O2	-3.112	0.000	-0.000	NH3	-3.598	-0.000	0.000
	-0.000	-4.397	-0.000		0.000	-2.983	-0.103		-0.000	-3.808	0.000
	0.000	-0.000	0.213		-0.000	-0.103	-5.205		0.000	0.000	-3.808
CH+	8.437	-0.000	-0.000	H2S	-5.628	0.000	0.000	OCS	-6.893	0.000	-0.000
	-0.000	8.437	-0.000		0.000	-6.242	-0.000		0.000	-6.893	0.000
	-0.000	-0.000	-1.443		0.000	-0.000	-5.792		-0.000	0.000	-8.739
CH2O	-3.087	-0.000	0.000	H4C2O	-8.195	-0.000	0.000	OF2	-2.131	-0.000	0.000
	-0.000	-1.935	-0.000		-0.000	-5.539	-0.000		-0.000	-4.049	0.000
	0.000	-0.000	0.048		0.000	-0.000	-6.899		0.000	0.000	-3.372
CH3F	-5.222	0.000	0.000	HCN	-3.067	0.000	0.000	PN	-2.931	-0.000	-0.000
	0.000	-3.427	0.000		0.000	-3.067	-0.000		-0.000	-2.931	0.000
	0.000	0.000	-3.427		0.000	-0.000	-4.376		-0.000	0.000	-6.589
CH4	-4.075	-0.000	-0.000	HCP	-5.839	0.000	-0.000	SO2	-4.813	-0.000	-0.000
	-0.000	-4.075	0.000		0.000	-5.839	-0.000		-0.000	-3.839	-0.000
	-0.000	0.000	-4.076		-0.000	-0.000	-7.434		-0.000	-0.000	-3.916
CO	-2.185	-0.000	-0.000	HF	-2.300	0.000	-0.000	SiH+	3.324	-0.000	0.000
	-0.000	-2.185	-0.000		0.000	-2.300	-0.000		-0.000	3.324	-0.000
	-0.000	-0.000	-3.844		-0.000	-0.000	-2.189		0.000	-0.000	-3.142

Table 3: χ computed with HF in aug-cc-pCVTZ basis

AlF	-4.688	-0.000	0.000	FCCH	-5.400	-0.000	-0.000	HFCO	-3.435	0.032	-0.000
	-0.000	-4.688	-0.000		-0.000	-5.400	-0.000		0.032	-3.616	-0.000
	0.000	-0.000	-5.837		-0.000	-0.000	-6.398		-0.000	-0.000	-4.799
AlH	3.484	-0.000	-0.000	FCN	-4.350	-0.000	0.000	HOF	-4.243	-0.022	-0.000
	-0.000	3.484	0.000		-0.000	-4.350	-0.000		-0.022	-2.328	-0.000
	-0.000	0.000	-4.632		0.000	-0.000	-5.681		-0.000	-0.000	-2.727
BH	7.040	-0.000	-0.000	FNO	-4.145	-2.020	-0.000	LiF	-2.324	-0.000	-0.000
	-0.000	7.040	-0.000		-2.020	-2.676	-0.000		-0.000	-2.324	-0.000
	-0.000	-0.000	-2.511		-0.000	-0.000	-1.262		-0.000	-0.000	-2.614
BeH-	3.986	0.000	-0.000	H2	-0.876	0.000	-0.000	LiH	-1.408	-0.000	-0.000
	0.000	3.986	-0.000		0.000	-0.876	-0.000		-0.000	-1.408	-0.000
	-0.000	-0.000	-7.601		-0.000	-0.000	-0.777		-0.000	-0.000	-1.950
C2H4	-3.545	0.000	0.000	H2C2O	-5.134	-0.000	-0.000	N2	-1.924	-0.000	-0.000
	0.000	-4.277	0.000		-0.000	-5.503	0.000		-0.000	-1.924	0.000
	0.000	0.000	-5.666		-0.000	0.000	-5.815		-0.000	0.000	-3.863
C3H4	-8.625	0.000	0.000	H2O	-2.954	-0.000	-0.000	N2O	-3.649	0.000	0.000
	0.000	-4.165	0.000		-0.000	-2.901	-0.000		0.000	-3.649	0.000
	0.000	0.000	-5.403		-0.000	-0.000	-2.938		0.000	0.000	-5.732
C4H4	-5.078	0.000	0.000	H2O2	-3.341	0.000	0.000	NH3	-3.506	-0.000	0.000
	0.000	-4.419	0.000		0.000	-3.256	-0.097		-0.000	-3.713	0.000
	0.000	0.000	0.435		0.000	-0.097	-5.033		0.000	0.000	-3.713
CH+	10.452	-0.000	0.000	H2S	-5.416	0.000	0.000	OCS	-6.958	0.000	-0.000
	-0.000	10.452	0.000		0.000	-6.199	-0.000		0.000	-6.958	0.000
	0.000	0.000	-1.443		0.000	-0.000	-5.630		-0.000	0.000	-8.807
CH2O	-3.416	-0.000	0.000	H4C2O	-8.096	-0.000	0.000	OF2	-2.454	-0.000	0.000
	-0.000	-1.782	-0.000		-0.000	-5.671	-0.000		-0.000	-4.296	0.000
	0.000	-0.000	-0.112		0.000	-0.000	-6.954		0.000	0.000	-3.575
CH3F	-5.115	0.000	0.000	HCN	-3.100	0.000	0.000	PN	-2.446	-0.000	-0.000
	0.000	-3.491	0.000		0.000	-3.100	-0.000		-0.000	-2.446	0.000
	0.000	0.000	-3.491		0.000	-0.000	-4.453		-0.000	0.000	-6.660
CH4	-3.977	0.000	-0.000	HCP	-5.930	0.000	-0.000	SO2	-4.909	0.000	0.000
	0.000	-3.977	0.000		0.000	-5.930	-0.000		0.000	-2.841	-0.000
	-0.000	0.000	-3.977		-0.000	-0.000	-7.605		0.000	-0.000	-3.767
CO	-1.990	-0.000	-0.000	HF	-2.226	0.000	-0.000	SiH+	3.411	-0.000	-0.000
	-0.000	-1.990	0.000		0.000	-2.226	-0.000		-0.000	3.411	-0.000
	-0.000	0.000	-3.801		-0.000	-0.000	-2.115		-0.000	-0.000	-3.154

Table 4: χ computed with LDA in aug-cc-pCVTZ basis

AlF	-4.566	0.000	0.000	FCCH	-5.146	0.000	-0.000	HFCO	-3.279	-0.059	-0.000
	0.000	-4.566	-0.000		0.000	-5.146	0.000		-0.059	-3.347	-0.000
	0.000	-0.000	-5.952		-0.000	0.000	-6.397		-0.000	-0.000	-4.673
AlH	4.460	-0.000	-0.000	FCN	-4.095	-0.000	0.000	HOF	-4.416	-0.002	0.000
	-0.000	4.460	-0.000		-0.000	-4.095	0.000		-0.002	-1.928	0.000
	-0.000	-0.000	-4.632		0.000	0.000	-5.710		0.000	0.000	-2.367
BH	7.512	-0.000	0.000	FNO	x	x	x	LiF	-2.317	-0.000	-0.000
	-0.000	7.512	-0.000		x	x	x		-0.000	-2.317	-0.000
	0.000	-0.000	-2.579		x	x	x		-0.000	-0.000	-2.841
BeH-	4.631	0.000	-0.000	H2	-0.916	-0.000	0.000	LiH	-1.515	0.000	-0.000
	0.000	4.631	-0.000		-0.000	-0.916	-0.000		0.000	-1.515	0.000
	-0.000	-0.000	-8.638		0.000	-0.000	-0.807		-0.000	0.000	-2.136
C2H4	-3.299	-0.000	-0.000	H2C2O	-5.060	-0.000	0.000	N2	-1.896	0.000	0.000
	-0.000	-3.751	-0.000		-0.000	-5.251	-0.000		0.000	-1.896	0.000
	-0.000	-0.000	-5.542		0.000	-0.000	-5.955		0.000	0.000	-3.854
C3H4	-8.449	0.000	0.000	H2O	-3.074	-0.000	-0.000	N2O	-3.495	-0.000	0.000
	0.000	-3.848	-0.000		-0.000	-3.028	-0.000		-0.000	-3.495	0.000
	0.000	-0.000	-5.375		-0.000	-0.000	-3.067		0.000	0.000	-5.724
C4H4	-4.852	-0.000	0.000	H2O2	-2.894	-0.000	0.000	NH3	-3.642	-0.000	0.000
	-0.000	-4.006	0.000		-0.000	-2.729	-0.127		-0.000	-3.849	0.000
	0.000	0.000	0.903		0.000	-0.127	-5.219		0.000	0.000	-3.849
CH+	10.154	-0.000	0.000	H2S	-5.667	0.000	-0.000	OCS	-6.625	-0.000	-0.000
	-0.000	10.154	0.000		0.000	-6.260	0.000		-0.000	-6.625	0.000
	0.000	0.000	-1.471		-0.000	0.000	-5.822		-0.000	0.000	-8.686
CH2O	-2.972	0.000	0.000	H4C2O	-8.089	-0.000	-0.000	OF2	-1.817	0.000	0.000
	0.000	-1.667	-0.000		-0.000	-5.332	0.000		0.000	-3.617	-0.000
	0.000	-0.000	0.977		-0.000	0.000	-6.729		0.000	-0.000	-2.942
CH3F	-5.300	0.000	0.000	HCN	-2.835	0.000	-0.000	PN	-2.129	-0.000	0.000
	0.000	-3.351	0.000		0.000	-2.835	-0.000		-0.000	-2.129	0.000
	0.000	0.000	-3.351		-0.000	-0.000	-4.414		0.000	0.000	-6.577
CH4	-4.178	-0.000	-0.000	HCP	-5.373	-0.000	-0.000	SO2	-4.673	0.000	-0.000
	-0.000	-4.178	0.000		-0.000	-5.373	-0.000		0.000	-3.101	0.000
	-0.000	0.000	-4.178		-0.000	-0.000	-7.426		-0.000	0.000	-3.483
CO	-1.997	-0.000	-0.000	HF	-2.335	0.000	-0.000	SiH+	4.245	-0.000	0.000
	-0.000	-1.997	-0.000		0.000	-2.335	0.000		-0.000	4.245	0.000
	-0.000	-0.000	-3.868		-0.000	0.000	-2.226		0.000	0.000	-3.161

Table 5: χ computed with KT3 in aug-cc-pCVTZ basis

AlF	-4.567	-0.000	-0.000	FCCH	-5.146	-0.000	-0.000	HFCO	-3.365	-0.027	-0.000
	-0.000	-4.567	0.000		-0.000	-5.146	0.000		-0.027	-3.379	0.000
	-0.000	0.000	-5.880		-0.000	0.000	-6.330		-0.000	0.000	-4.573
AlH	3.216	-0.000	0.000	FCN	-4.122	-0.000	-0.000	HOF	-4.301	0.010	0.000
	-0.000	3.216	0.000		-0.000	-4.122	0.000		0.010	-1.929	0.000
	0.000	0.000	-4.578		-0.000	0.000	-5.642		0.000	0.000	-2.329
BH	5.389	-0.000	0.000	FNO	-4.000	-2.270	0.000	LiF	-2.294	-0.000	0.000
	-0.000	5.389	-0.000		-2.270	-2.344	0.000		-0.000	-2.294	0.000
	0.000	-0.000	-2.502		0.000	0.000	-0.758		0.000	0.000	-2.792
BeH-	4.995	-0.000	-0.000	H2	-0.877	0.000	0.000	LiH	-1.599	-0.000	-0.000
	-0.000	4.995	0.000		0.000	-0.877	-0.000		-0.000	-1.599	0.000
	-0.000	0.000	-8.426		0.000	-0.000	-0.768		-0.000	0.000	-2.047
C2H4	-3.409	0.000	-0.000	H2C2O	-4.918	0.000	0.000	N2	-1.919	-0.000	0.000
	0.000	-3.873	0.000		0.000	-5.181	-0.000		-0.000	-1.919	0.000
	-0.000	0.000	-5.360		0.000	-0.000	-5.753		0.000	0.000	-3.805
C3H4	-8.131	-0.000	-0.000	H2O	-2.972	-0.000	0.000	N2O	-3.407	-0.000	0.000
	-0.000	-3.781	0.000		-0.000	-2.945	0.000		-0.000	-3.407	0.000
	-0.000	0.000	-5.333		0.000	0.000	-2.983		0.000	0.000	-5.651
C4H4	-5.067	-0.000	0.000	H2O2	-2.830	0.000	0.000	NH3	-3.490	-0.000	0.000
	-0.000	-4.083	0.000		0.000	-2.676	-0.116		-0.000	-3.714	0.000
	0.000	0.000	1.024		0.000	-0.116	-5.069		0.000	0.000	-3.714
CH+	6.276	-0.000	0.000	H2S	-5.436	-0.000	-0.000	OCS	-6.569	0.000	0.000
	-0.000	6.276	0.000		-0.000	-6.109	0.000		0.000	-6.569	0.000
	0.000	0.000	-1.435		-0.000	0.000	-5.616		0.000	0.000	-8.599
CH2O	-2.964	0.000	0.000	H4C2O	-7.867	0.000	-0.000	OF2	-1.849	-0.000	0.000
	0.000	-1.728	-0.000		0.000	-5.249	-0.000		-0.000	-3.654	-0.000
	0.000	-0.000	0.201		-0.000	-0.000	-6.526		0.000	-0.000	-2.998
CH3F	-5.067	0.000	-0.000	HCN	-2.894	-0.000	0.000	PN	-2.277	-0.000	-0.000
	0.000	-3.275	0.000		-0.000	-2.894	-0.000		-0.000	-2.277	-0.000
	-0.000	0.000	-3.275		0.000	-0.000	-4.365		-0.000	-0.000	-6.521
CH4	-3.953	0.000	0.000	HCP	-5.521	0.000	-0.000	SO2	-4.646	-0.000	0.000
	0.000	-3.953	0.000		0.000	-5.521	-0.000		-0.000	-3.076	0.000
	0.000	0.000	-3.953		-0.000	-0.000	-7.365		0.000	0.000	-3.442
CO	-2.016	0.000	0.000	HF	-2.273	-0.000	-0.000	SiH+	3.265	-0.000	-0.000
	0.000	-2.016	0.000		-0.000	-2.273	0.000		-0.000	3.265	-0.000
	0.000	0.000	-3.805		-0.000	0.000	-2.175		-0.000	-0.000	-3.115

Table 6: χ computed with M06 in aug-cc-pCVTZ basis

AlF	-5.005	-0.000	-0.000	FCCH	-5.297	-0.000	-0.000	HFCO	-3.640	0.010	0.000
	-0.000	-5.005	-0.000		-0.000	-5.297	-0.000		0.010	-3.582	0.000
	-0.000	-0.000	-6.561		-0.000	-0.000	-7.002		0.000	0.000	-4.875
AlH	5.539	-0.000	-0.000	FCN	-4.270	-0.000	-0.000	HOF	-4.843	-0.024	-0.000
	-0.000	5.539	-0.000		-0.000	-4.270	0.000		-0.024	-1.931	0.000
	-0.000	-0.000	-5.168		-0.000	0.000	-6.197		-0.000	0.000	-2.356
BH	7.012	0.000	-0.000	FNO	x	x	x	LiF	-2.313	-0.000	-0.000
	0.000	7.012	-0.000		x	x	x		-0.000	-2.313	0.000
	-0.000	-0.000	-2.901		x	x	x		-0.000	0.000	-3.254
BeH-	x	x	x	H2	-1.000	-0.000	0.000	LiH	-2.213	-0.000	0.000
	x	x	x		-0.000	-1.000	0.000		-0.000	-2.213	-0.000
	x	x	x		0.000	0.000	-0.881		0.000	-0.000	-2.592
C2H4	-3.516	0.000	0.000	H2C2O	-5.219	-0.000	0.000	N2	-2.081	0.000	-0.000
	0.000	-4.060	0.000		-0.000	-5.233	0.000		0.000	-2.081	0.000
	0.000	0.000	-5.632		0.000	0.000	-6.767		-0.000	0.000	-4.187
C3H4	-8.918	-0.000	0.000	H2O	-3.339	-0.000	-0.000	N2O	-3.446	-0.000	-0.000
	-0.000	-3.567	-0.000		-0.000	-3.335	-0.000		-0.000	-3.446	-0.000
	0.000	-0.000	-5.680		-0.000	-0.000	-3.347		-0.000	-0.000	-6.191
C4H4	-5.176	-0.000	0.000	H2O2	-2.777	0.000	0.000	NH3	-3.940	-0.000	0.000
	-0.000	-3.368	-0.000		0.000	-2.571	-0.217		-0.000	-4.176	0.000
	0.000	-0.000	1.573		0.000	-0.217	-5.709		0.000	0.000	-4.176
CH+	8.766	0.000	-0.000	H2S	-6.126	0.000	-0.000	OCS	-6.808	-0.000	0.000
	0.000	8.766	0.000		0.000	-6.821	0.000		-0.000	-6.808	-0.000
	-0.000	0.000	-1.585		-0.000	0.000	-6.329		0.000	-0.000	-9.436
CH2O	-2.459	-0.000	0.000	H4C2O	-8.639	-0.000	0.000	OF2	-1.969	-0.000	-0.000
	-0.000	-1.694	0.000		-0.000	-5.212	0.000		-0.000	-3.807	-0.000
	0.000	0.000	1.252		0.000	0.000	-6.926		-0.000	-0.000	-3.055
CH3F	-5.645	0.000	0.000	HCN	-2.964	0.000	-0.000	PN	-2.027	-0.000	0.000
	0.000	-3.215	0.000		0.000	-2.964	-0.000		-0.000	-2.027	-0.000
	0.000	0.000	-3.215		-0.000	-0.000	-4.790		0.000	-0.000	-7.134
CH4	-4.484	-0.000	-0.000	HCP	-5.627	-0.000	0.000	SO2	-4.976	0.000	-0.000
	-0.000	-4.484	0.000		-0.000	-5.627	0.000		0.000	-3.798	0.000
	-0.000	0.000	-4.484		0.000	0.000	-8.069		-0.000	0.000	-3.829
CO	-2.282	-0.000	0.000	HF	-2.561	-0.000	-0.000	SiH+	x	x	x
	-0.000	-2.282	-0.000		-0.000	-2.561	-0.000		x	x	x
	0.000	-0.000	-4.226		-0.000	-0.000	-2.459		x	x	x

Table 7: χ computed with TPSS in aug-cc-pCVTZ basis

AlF	-4.562	0.000	0.000	FCCH	-5.161	0.000	0.000	HFCO	-3.327	-0.031	0.000
	0.000	-4.562	-0.000		0.000	-5.161	0.000		-0.031	-3.361	-0.000
	0.000	-0.000	-5.919		0.000	0.000	-6.341		0.000	-0.000	-4.656
AlH	2.991	0.000	0.000	FCN	-4.120	0.000	-0.000	HOF	-4.346	0.005	-0.000
	0.000	2.991	0.000		0.000	-4.120	-0.000		0.005	-1.993	-0.000
	0.000	0.000	-4.576		-0.000	-0.000	-5.660		-0.000	-0.000	-2.390
BH	5.323	-0.000	-0.000	FNO	-3.310	-2.921	0.000	LiF	-2.316	-0.000	0.000
	-0.000	5.323	-0.000		-2.921	-1.111	0.000		-0.000	-2.316	-0.000
	-0.000	-0.000	-2.495		0.000	0.000	0.894		0.000	-0.000	-2.806
BeH-	2.964	-0.000	-0.000	H2	-0.872	0.000	-0.000	LiH	-1.452	0.000	0.000
	-0.000	2.964	0.000		0.000	-0.872	0.000		0.000	-1.452	-0.000
	-0.000	0.000	-7.931		-0.000	0.000	-0.765		0.000	-0.000	-1.998
C2H4	-3.361	0.000	-0.000	H2C2O	-4.939	0.000	-0.000	N2	-1.863	-0.000	0.000
	0.000	-3.878	0.000		0.000	-5.242	-0.000		-0.000	-1.863	0.000
	-0.000	0.000	-5.460		-0.000	-0.000	-5.773		0.000	0.000	-3.809
C3H4	-8.289	-0.000	-0.000	H2O	-2.998	0.000	0.000	N2O	-3.469	-0.000	0.000
	-0.000	-3.945	0.000		0.000	-2.973	-0.000		-0.000	-3.469	0.000
	-0.000	0.000	-5.318		0.000	-0.000	-3.007		0.000	0.000	-5.672
C4H4	-4.941	0.000	0.000	H2O2	-2.913	0.000	-0.000	NH3	-3.512	-0.000	-0.000
	0.000	-4.195	-0.000		0.000	-2.766	-0.115		-0.000	-3.740	-0.000
	0.000	-0.000	0.509		-0.000	-0.115	-5.121		-0.000	-0.000	-3.740
CH+	6.766	0.000	-0.000	H2S	-5.441	-0.000	-0.000	OCS	-6.619	-0.000	0.000
	0.000	6.766	0.000		-0.000	-6.136	-0.000		-0.000	-6.619	0.000
	-0.000	0.000	-1.437		-0.000	-0.000	-5.634		0.000	0.000	-8.635
CH2O	-3.081	-0.000	0.000	H4C2O	-7.934	-0.000	0.000	OF2	-1.889	-0.000	0.000
	-0.000	-1.694	0.000		-0.000	-5.372	-0.000		-0.000	-3.729	-0.000
	0.000	0.000	0.300		0.000	-0.000	-6.640		0.000	-0.000	-3.062
CH3F	-5.107	0.000	-0.000	HCN	-2.852	-0.000	-0.000	PN	-2.135	-0.000	-0.000
	0.000	-3.356	0.000		-0.000	-2.852	-0.000		-0.000	-2.135	0.000
	-0.000	0.000	-3.356		-0.000	-0.000	-4.365		-0.000	0.000	-6.534
CH4	-3.970	0.000	-0.000	HCP	-5.473	0.000	0.000	SO2	-4.678	0.000	-0.000
	0.000	-3.970	-0.000		0.000	-5.473	0.000		0.000	-2.998	0.000
	-0.000	-0.000	-3.971		0.000	0.000	-7.376		-0.000	0.000	-3.421
CO	-1.976	-0.000	0.000	HF	-2.298	0.000	-0.000	SiH+	3.120	-0.000	0.000
	-0.000	-1.976	-0.000		0.000	-2.298	-0.000		-0.000	3.120	-0.000
	0.000	-0.000	-3.813		-0.000	-0.000	-2.197		0.000	-0.000	-3.123

Table 8: $\mathcal{A}$ computed with CCSD in aug-cc-pCVTZ basis

AlH	-9.956	0.000	0.000	H2S	-3.864	0.000	0.000
	0.000	-9.956	0.000		0.000	-5.880	-0.000
	0.000	0.000	-4.051		0.000	-0.000	-5.331
BH	-4.282	-0.001	0.000	HF	-1.050	-0.000	0.000
	-0.001	-4.281	0.000		-0.000	-1.050	-0.000
	0.000	0.000	-1.841		0.000	-0.000	-1.111
BeH-	-71.119	0.000	0.000	HOF	-2.030	0.200	-0.000
	0.000	-71.119	-0.000		0.200	-2.098	-0.000
	0.000	-0.000	-42.056		-0.000	-0.000	0.155
CH+	-1.163	0.000	0.000	LiF	-2.434	-0.000	-0.000
	0.000	-1.163	0.000		-0.000	-2.434	0.000
	0.000	0.000	-0.637		-0.000	0.000	-1.705
CO	-4.856	-0.002	0.000	LiH	-6.460	0.000	0.000
	-0.002	-4.856	0.000		0.000	-6.459	0.000
	0.000	0.000	-2.857		0.000	0.000	-3.538
FNO	-0.571	-5.587	0.083	N2	-4.714	0.000	0.000
	-5.587	3.004	-0.128		0.000	-4.714	0.000
	0.083	-0.128	6.736		0.000	0.000	-2.790
H2	-0.781	-0.000	-0.000	NH3	-2.292	-0.000	0.000
	-0.000	-0.781	-0.000		-0.000	-3.153	0.000
	-0.000	-0.000	-0.753		0.000	0.000	-3.153
H2O	-1.474	-0.000	-0.000	SiH+	-4.722	-0.000	-0.000
	-0.000	-1.983	0.000		-0.000	-4.722	-0.000
	-0.000	0.000	-1.852		-0.000	-0.000	-1.542

Table 9: $\mathcal{A}$ computed with MP2 in aug-cc-pCVTZ basis

AlF	-15.714	0.000	0.000	FCCH	-18.625	0.000	0.000	HFCO	-4.810	-1.301	-0.000
	0.000	-15.714	0.000		0.000	-18.625	0.000		-1.301	-11.251	-0.000
	0.000	0.000	-6.779		0.000	0.000	-4.796		-0.000	-0.000	-13.429
AlH	-10.360	-0.000	0.000	FCN	-12.864	0.000	-0.000	HOF	-2.109	0.190	-0.000
	-0.000	-10.360	-0.000		0.000	-12.864	0.000		0.190	-2.088	-0.000
	0.000	-0.000	-4.091		-0.000	0.000	-3.269		-0.000	-0.000	0.274
BH	-4.438	0.000	-0.000	FNO	1.964	-7.043	-0.000	LiF	-2.541	-0.000	-0.000
	0.000	-4.438	-0.000		-7.043	7.599	-0.000		-0.000	-2.541	-0.000
	-0.000	-0.000	-1.885		-0.000	-0.000	12.613		-0.000	-0.000	-1.824
BeH-	-79.415	-0.000	0.000	H2	-0.787	-0.000	-0.000	LiH	-6.390	-0.000	-0.000
	-0.000	-79.415	-0.000		-0.000	-0.787	-0.000		-0.000	-6.390	-0.000
	0.000	-0.000	-46.451		-0.000	-0.000	-0.761		-0.000	-0.000	-3.431
C2H4	-12.125	0.000	0.000	H2C2O	-21.591	-0.000	-0.000	N2	-4.808	-0.000	-0.000
	0.000	-6.760	-0.000		-0.000	-21.012	0.000		-0.000	-4.808	0.000
	0.000	-0.000	-14.220		-0.000	0.000	-5.534		-0.000	0.000	-2.821
C3H4	-18.137	0.000	-0.000	H2O	-1.515	-0.000	-0.000	N2O	-12.502	-0.000	-0.000
	0.000	-14.822	-0.000		-0.000	-2.097	-0.000		-0.000	-12.502	-0.000
	-0.000	-0.000	-10.441		-0.000	-0.000	-1.932		-0.000	-0.000	-2.561
C4H4	-17.365	-0.001	0.000	H2O2	-2.021	0.000	0.000	NH3	-2.319	-0.000	0.000
	-0.001	-24.407	0.000		0.000	-3.930	0.404		-0.000	-3.285	0.000
	0.000	0.000	-27.064		0.000	0.404	-2.699		0.000	0.000	-3.285
CH+	-1.122	-0.000	-0.000	H2S	-3.896	-0.000	0.000	OCS	-23.408	0.000	-0.000
	-0.000	-1.122	-0.000		-0.000	-6.047	-0.000		0.000	-23.408	0.000
	-0.000	-0.000	-0.642		0.000	-0.000	-5.461		-0.000	0.000	-5.996
CH2O	-8.558	-0.000	-0.000	H4C2O	-13.871	0.000	0.000	OF2	-1.026	0.000	-0.000
	-0.000	-5.458	-0.000		0.000	-9.278	-0.000		0.000	-2.907	-0.000
	-0.000	-0.000	-3.283		0.000	-0.000	-10.708		-0.000	-0.000	-4.861
CH3F	-4.050	0.000	0.000	HCN	-7.131	-0.000	-0.000	PN	-11.898	-0.000	0.000
	0.000	-7.293	-0.000		-0.000	-7.131	-0.000		-0.000	-11.898	0.000
	0.000	-0.000	-7.293		-0.000	-0.000	-3.795		0.000	0.000	-6.352
CH4	-4.469	0.000	-0.000	HCP	-16.037	-0.000	0.000	SO2	-18.378	-0.000	0.000
	0.000	-4.469	-0.000		-0.000	-16.037	0.000		-0.000	-6.569	-0.000
	-0.000	-0.000	-4.469		0.000	0.000	-8.247		0.000	-0.000	-15.718
CO	-5.054	0.000	-0.000	HF	-1.095	-0.000	-0.000	SiH+	-4.824	0.000	-0.000
	0.000	-5.054	-0.000		-0.000	-1.095	-0.000		0.000	-4.824	-0.000
	-0.000	-0.000	-2.966		-0.000	-0.000	-1.168		-0.000	-0.000	-1.538

Table 10: $\mathcal{A}$ computed with HF in aug-cc-pCVTZ basis

AlF	-16.064	0.000	0.000	FCCH	-18.464	0.000	0.000	HFCO	-4.589	-1.265	-0.000
	0.000	-16.064	0.000		0.000	-18.464	0.000		-1.265	-11.023	-0.000
	0.000	0.000	-6.731		0.000	0.000	-4.894		-0.000	-0.000	-12.966
AlH	-11.091	-0.000	0.000	FCN	-12.675	0.000	-0.000	HOF	-1.840	0.227	-0.000
	-0.000	-11.091	-0.000		0.000	-12.675	0.000		0.227	-1.956	-0.000
	0.000	-0.000	-4.487		-0.000	0.000	-3.365		-0.000	-0.000	0.372
BH	-4.426	0.000	0.000	FNO	10.871	-19.439	-0.000	LiF	-2.271	-0.000	-0.000
	0.000	-4.426	-0.000		-19.439	25.339	-0.000		-0.000	-2.271	0.000
	0.000	-0.000	-1.927		-0.000	-0.000	39.057		-0.000	0.000	-1.491
BeH-	-78.365	-0.000	0.000	H2	-0.799	0.000	-0.000	LiH	-6.412	-0.000	0.000
	-0.000	-78.365	0.000		0.000	-0.799	0.000		-0.000	-6.412	-0.000
	0.000	0.000	-46.470		-0.000	0.000	-0.776		0.000	-0.000	-3.362
C2H4	-12.022	0.000	0.000	H2C2O	-21.586	-0.000	0.000	N2	-4.656	0.000	-0.000
	0.000	-6.887	-0.000		-0.000	-20.617	0.000		0.000	-4.656	0.000
	0.000	-0.000	-14.031		0.000	0.000	-5.481		-0.000	0.000	-2.859
C3H4	-17.817	0.000	-0.000	H2O	-1.445	-0.000	-0.000	N2O	-12.289	0.000	-0.000
	0.000	-15.055	-0.000		-0.000	-1.807	-0.000		0.000	-12.289	-0.000
	-0.000	-0.000	-10.451		-0.000	-0.000	-1.743		-0.000	-0.000	-2.586
C4H4	-16.945	-0.001	-0.000	H2O2	-2.121	0.000	0.000	NH3	-2.335	-0.000	0.000
	-0.001	-25.075	0.000		0.000	-3.761	0.467		-0.000	-2.993	0.000
	-0.000	0.000	-27.214		0.000	0.467	-2.411		0.000	0.000	-2.993
CH+	-1.015	-0.000	-0.000	H2S	-3.858	-0.000	-0.000	OCS	-23.710	0.000	-0.000
	-0.000	-1.015	-0.000		-0.000	-5.864	-0.000		0.000	-23.710	0.000
	-0.000	-0.000	-0.649		-0.000	-0.000	-5.345		-0.000	0.000	-6.088
CH2O	-8.184	-0.000	-0.000	H4C2O	-14.050	0.000	0.000	OF2	-1.452	0.000	-0.000
	-0.000	-5.624	-0.000		0.000	-8.992	-0.000		0.000	-2.425	-0.000
	-0.000	-0.000	-3.359		0.000	-0.000	-10.883		-0.000	-0.000	-4.731
CH3F	-3.985	0.000	0.000	HCN	-6.961	0.000	-0.000	PN	-11.372	-0.000	0.000
	0.000	-7.200	-0.000		0.000	-6.961	0.000		-0.000	-11.372	0.000
	0.000	-0.000	-7.200		-0.000	0.000	-3.933		0.000	0.000	-6.500
CH4	-4.350	0.000	-0.000	HCP	-16.003	-0.000	0.000	SO2	-17.931	-0.000	0.000
	0.000	-4.350	-0.000		-0.000	-16.003	0.000		-0.000	-6.039	-0.000
	-0.000	-0.000	-4.350		0.000	0.000	-8.705		0.000	-0.000	-15.348
CO	-4.677	0.000	-0.000	HF	-0.972	-0.000	-0.000	SiH+	-4.981	0.000	0.000
	0.000	-4.677	-0.000		-0.000	-0.972	0.000		0.000	-4.981	-0.000
	-0.000	-0.000	-2.792		-0.000	0.000	-1.005		0.000	-0.000	-1.579

Table 11: $\mathcal{A}$ computed with LDA in aug-cc-pCVTZ basis

AlF	-15.543	0.000	0.000	FCCH	-18.539	0.000	0.000	HFCO	-4.880	-1.330	0.000
	0.000	-15.543	0.000		0.000	-18.539	0.000		-1.330	-11.184	0.000
	0.000	0.000	-6.856		0.000	0.000	-4.878		0.000	0.000	-13.345
AlH	-10.627	0.000	0.000	FCN	-12.539	-0.000	-0.000	HOF	-2.151	0.208	0.000
	0.000	-10.627	0.000		-0.000	-12.539	-0.000		0.208	-1.653	-0.000
	0.000	0.000	-3.969		-0.000	-0.000	-3.282		0.000	-0.000	1.285
BH	-4.640	0.000	-0.000	FNO	x	x	x	LiF	-2.600	0.000	0.000
	0.000	-4.640	-0.000		x	x	x		0.000	-2.600	0.000
	-0.000	-0.000	-2.033		x	x	x		0.000	0.000	-2.008
BeH-	-134.053	0.000	0.000	H2	-0.910	-0.000	0.000	LiH	-7.170	-0.000	0.000
	0.000	-134.053	0.000		-0.000	-0.910	0.000		-0.000	-7.170	0.000
	0.000	0.000	-77.614		0.000	0.000	-0.877		0.000	0.000	-4.126
C2H4	-12.160	-0.000	-0.000	H2C2O	-21.187	-0.000	-0.000	N2	-4.845	-0.000	-0.000
	-0.000	-6.662	0.000		-0.000	-20.880	-0.000		-0.000	-4.845	-0.000
	-0.000	0.000	-14.354		-0.000	-0.000	-5.478		-0.000	-0.000	-2.887
C3H4	-17.860	0.000	-0.000	H2O	-1.510	0.000	-0.000	N2O	-12.291	-0.000	-0.000
	0.000	-14.736	-0.000		0.000	-2.197	-0.000		-0.000	-12.291	-0.000
	-0.000	-0.000	-10.356		-0.000	-0.000	-1.998		-0.000	-0.000	-2.550
C4H4	-17.297	-0.000	-0.000	H2O2	-1.211	-0.000	-0.000	NH3	-2.279	-0.000	-0.000
	-0.000	-24.067	-0.000		-0.000	-3.515	0.405		-0.000	-3.416	-0.000
	-0.000	-0.000	-26.147		-0.000	0.405	-2.688		-0.000	-0.000	-3.416
CH+	-1.048	0.000	-0.000	H2S	-3.786	-0.000	-0.000	OCS	-22.803	0.000	-0.000
	0.000	-1.048	-0.000		-0.000	-6.165	0.000		0.000	-22.803	-0.000
	-0.000	-0.000	-0.674		-0.000	0.000	-5.530		-0.000	-0.000	-5.843
CH2O	-8.667	0.000	0.000	H4C2O	-13.418	0.000	0.000	OF2	1.043	0.000	-0.000
	0.000	-5.326	-0.000		0.000	-9.278	-0.000		0.000	-3.042	-0.000
	0.000	-0.000	-3.248		0.000	-0.000	-10.764		-0.000	-0.000	-4.559
CH3F	-4.082	0.000	0.000	HCN	-7.172	-0.000	-0.000	PN	-11.565	-0.000	0.000
	0.000	-7.200	-0.000		-0.000	-7.172	-0.000		-0.000	-11.565	-0.000
	0.000	-0.000	-7.200		-0.000	-0.000	-3.851		0.000	-0.000	-6.218
CH4	-4.602	-0.000	-0.000	HCP	-15.903	0.000	0.000	SO2	-17.903	-0.000	0.000
	-0.000	-4.602	-0.000		0.000	-15.903	0.000		-0.000	-6.422	-0.000
	-0.000	-0.000	-4.602		0.000	0.000	-8.151		0.000	-0.000	-15.234
CO	-5.030	0.000	0.000	HF	-1.158	0.000	-0.000	SiH+	-4.762	-0.000	-0.000
	0.000	-5.030	-0.000		0.000	-1.158	-0.000		-0.000	-4.762	0.000
	0.000	-0.000	-3.003		-0.000	-0.000	-1.250		-0.000	0.000	-1.500

Table 12: $\mathcal{A}$ computed with KT3 in aug-cc-pCVTZ basis

AlF	-15.649	-0.000	-0.000	FCCH	-18.379	0.000	0.000	HFCO	-4.799	-1.287	-0.000
	-0.000	-15.649	-0.000		0.000	-18.379	-0.000		-1.287	-10.926	-0.000
	-0.000	-0.000	-6.991		0.000	-0.000	-4.896		-0.000	-0.000	-13.220
AlH	-10.658	0.000	0.000	FCN	-12.507	0.000	-0.000	HOF	-2.110	0.198	0.000
	0.000	-10.658	0.000		0.000	-12.507	-0.000		0.198	-1.923	-0.000
	0.000	0.000	-4.128		-0.000	-0.000	-3.268		0.000	-0.000	0.550
BH	-4.847	-0.000	-0.000	FNO	10.283	-18.453	-0.000	LiF	-2.504	0.000	0.000
	-0.000	-4.847	0.000		-18.453	24.442	-0.000		0.000	-2.504	-0.000
	-0.000	0.000	-1.822		-0.000	-0.000	37.518		0.000	-0.000	-2.038
BeH-	-145.209	0.000	-0.000	H2	-0.857	0.000	0.000	LiH	-6.970	-0.000	0.000
	0.000	-145.209	0.000		0.000	-0.857	-0.000		-0.000	-6.970	-0.000
	-0.000	0.000	-69.255		0.000	-0.000	-0.827		0.000	-0.000	-4.081
C2H4	-11.835	0.000	-0.000	H2C2O	-20.850	0.000	-0.000	N2	-4.810	0.000	-0.000
	0.000	-6.671	0.000		0.000	-20.361	0.000		0.000	-4.810	-0.000
	-0.000	0.000	-14.064		-0.000	0.000	-5.375		-0.000	-0.000	-2.884
C3H4	-17.278	-0.000	-0.000	H2O	-1.459	0.000	-0.000	N2O	-12.161	-0.000	-0.000
	-0.000	-14.171	0.000		0.000	-2.160	0.000		-0.000	-12.161	-0.000
	-0.000	0.000	-9.830		-0.000	0.000	-1.958		-0.000	-0.000	-2.474
C4H4	-16.602	0.000	-0.000	H2O2	-1.586	-0.000	-0.000	NH3	-2.137	-0.000	-0.000
	0.000	-23.776	0.000		-0.000	-3.628	0.415		-0.000	-3.331	0.000
	-0.000	0.000	-25.948		-0.000	0.415	-2.624		-0.000	0.000	-3.331
CH+	-1.267	-0.000	0.000	H2S	-3.696	-0.000	0.000	OCS	-22.721	-0.000	0.000
	-0.000	-1.267	0.000		-0.000	-6.096	0.000		-0.000	-22.721	0.000
	0.000	0.000	-0.627		0.000	0.000	-5.472		0.000	0.000	-5.813
CH2O	-8.500	0.000	-0.000	H4C2O	-12.940	0.000	-0.000	OF2	-0.379	0.000	0.000
	0.000	-5.141	-0.000		0.000	-8.881	0.000		0.000	-2.996	-0.000
	-0.000	-0.000	-3.212		-0.000	0.000	-10.356		0.000	-0.000	-4.579
CH3F	-3.989	0.000	-0.000	HCN	-7.100	0.000	0.000	PN	-11.503	0.000	0.000
	0.000	-6.957	0.000		0.000	-7.100	0.000		0.000	-11.503	-0.000
	-0.000	0.000	-6.957		0.000	0.000	-3.854		0.000	-0.000	-6.267
CH4	-4.411	0.000	0.000	HCP	-15.693	-0.000	0.000	SO2	-17.598	-0.000	0.000
	0.000	-4.411	-0.000		-0.000	-15.693	-0.000		-0.000	-6.364	-0.000
	0.000	-0.000	-4.411		0.000	-0.000	-8.224		0.000	-0.000	-14.921
CO	-5.010	-0.000	0.000	HF	-1.146	0.000	-0.000	SiH+	-4.739	-0.000	0.000
	-0.000	-5.010	0.000		0.000	-1.146	-0.000		-0.000	-4.739	-0.000
	0.000	0.000	-2.972		-0.000	-0.000	-1.230		0.000	-0.000	-1.505

Table 13: $\mathcal{A}$ computed with M06 in aug-cc-pCVTZ basis

AlF	-18.928	-0.000	-0.000	FCCH	-21.683	0.000	0.000	HFCO	-5.923	-1.682	-0.000
	-0.000	-18.928	0.000		0.000	-21.683	0.000		-1.682	-12.898	0.000
	-0.000	0.000	-9.003		0.000	0.000	-6.066		-0.000	0.000	-15.791
AlH	-14.314	0.000	0.000	FCN	-14.855	0.000	0.000	HOF	-2.679	0.224	-0.000
	0.000	-14.314	0.000		0.000	-14.855	0.000		0.224	-2.481	-0.000
	0.000	0.000	-3.830		0.000	0.000	-3.741		-0.000	-0.000	1.342
BH	-6.960	-0.000	-0.000	FNO	x	x	x	LiF	-2.977	-0.000	0.000
	-0.000	-6.960	0.000		x	x	x		-0.000	-2.977	0.000
	-0.000	0.000	-2.282		x	x	x		0.000	0.000	-3.029
BeH-	x	x	x	H2	-1.187	-0.000	-0.000	LiH	-9.939	-0.000	-0.000
	x	x	x		-0.000	-1.187	-0.000		-0.000	-9.939	0.000
	x	x	x		-0.000	-0.000	-1.127		-0.000	0.000	-6.392
C2H4	-14.159	0.000	0.000	H2C2O	-23.634	0.000	0.000	N2	-6.156	0.000	-0.000
	0.000	-8.049	0.000		0.000	-24.428	0.000		0.000	-6.156	0.000
	0.000	0.000	-16.587		0.000	0.000	-6.176		-0.000	0.000	-3.791
C3H4	-19.989	0.000	-0.000	H2O	-1.638	-0.000	0.000	N2O	-14.586	0.000	-0.000
	0.000	-16.838	0.000		-0.000	-2.926	-0.000		0.000	-14.586	-0.000
	-0.000	0.000	-10.710		0.000	-0.000	-2.547		-0.000	-0.000	-2.577
C4H4	-18.658	-0.000	0.000	H2O2	-1.243	0.000	-0.000	NH3	-2.297	-0.000	0.000
	-0.000	-28.101	-0.000		0.000	-4.471	0.474		-0.000	-4.511	0.000
	0.000	-0.000	-28.775		-0.000	0.474	-3.068		0.000	0.000	-4.511
CH+	-1.407	-0.000	0.000	H2S	-4.320	-0.000	0.000	OCS	-26.169	-0.000	-0.000
	-0.000	-1.407	-0.000		-0.000	-8.587	0.000		-0.000	-26.169	-0.000
	0.000	-0.000	-0.725		0.000	0.000	-7.630		-0.000	-0.000	-7.109
CH2O	-10.682	0.000	0.000	H4C2O	-14.276	-0.000	0.000	OF2	1.014	-0.000	0.000
	0.000	-5.375	-0.000		-0.000	-10.887	0.000		-0.000	-4.183	-0.000
	0.000	-0.000	-3.480		0.000	0.000	-11.594		0.000	-0.000	-6.014
CH3F	-4.543	0.000	0.000	HCN	-8.722	0.000	-0.000	PN	-14.497	-0.000	0.000
	0.000	-8.120	0.000		0.000	-8.722	0.000		-0.000	-14.497	-0.000
	0.000	0.000	-8.119		-0.000	0.000	-4.850		0.000	-0.000	-8.093
CH4	-5.460	0.000	0.000	HCP	-19.094	0.000	0.000	SO2	-21.539	0.000	0.000
	0.000	-5.460	-0.000		0.000	-19.094	-0.000		0.000	-8.235	-0.000
	0.000	-0.000	-5.460		0.000	-0.000	-10.620		0.000	-0.000	-18.244
CO	-6.501	0.000	-0.000	HF	-1.472	0.000	-0.000	SiH+	-5.465	0.000	0.000
	0.000	-6.501	-0.000		0.000	-1.472	0.000		0.000	-5.465	-0.000
	-0.000	-0.000	-3.955		-0.000	0.000	-1.660		0.000	-0.000	-1.478

Table 14: $\mathcal{A}$ computed with TPSS in aug-cc-pCVTZ basis

AlF	-15.548	0.000	-0.000	FCCH	-18.328	-0.000	0.000	HFCO	-4.786	-1.303	0.000
	0.000	-15.548	-0.000		-0.000	-18.328	-0.000		-1.303	-11.049	-0.000
	-0.000	-0.000	-6.772		0.000	-0.000	-4.804		0.000	-0.000	-13.204
AlH	-10.311	0.000	-0.000	FCN	-12.486	0.000	0.000	HOF	-2.103	0.204	-0.000
	0.000	-10.311	0.000		0.000	-12.486	-0.000		0.204	-1.883	0.000
	-0.000	0.000	-4.163		0.000	-0.000	-3.247		-0.000	0.000	0.658
BH	-4.538	-0.000	-0.000	FNO	36.472	-37.876	0.000	LiF	-2.545	-0.000	-0.000
	-0.000	-4.538	-0.000		-37.876	67.921	0.000		-0.000	-2.545	-0.000
	-0.000	-0.000	-1.932		0.000	0.000	94.009		-0.000	-0.000	-1.988
BeH-	-111.333	-0.000	0.000	H2	-0.820	-0.000	-0.000	LiH	-6.568	0.000	-0.000
	-0.000	-111.333	-0.000		-0.000	-0.820	-0.000		0.000	-6.568	0.000
	0.000	-0.000	-62.120		-0.000	-0.000	-0.790		-0.000	0.000	-3.685
C2H4	-11.837	0.000	-0.000	H2C2O	-20.949	0.000	-0.000	N2	-4.759	-0.000	-0.000
	0.000	-6.592	-0.000		0.000	-20.330	0.000		-0.000	-4.759	-0.000
	-0.000	-0.000	-14.025		-0.000	0.000	-5.367		-0.000	-0.000	-2.826
C3H4	-17.489	-0.000	0.000	H2O	-1.500	0.000	-0.000	N2O	-12.196	-0.000	0.000
	-0.000	-14.377	0.000		0.000	-2.140	-0.000		-0.000	-12.196	0.000
	0.000	0.000	-10.135		-0.000	-0.000	-1.959		0.000	0.000	-2.516
C4H4	-16.816	0.000	-0.000	H2O2	-1.635	-0.000	-0.000	NH3	-2.252	-0.000	0.000
	0.000	-23.731	0.000		-0.000	-3.673	0.423		-0.000	-3.303	0.000
	-0.000	0.000	-26.182		-0.000	0.423	-2.637		0.000	0.000	-3.303
CH+	-1.171	0.000	0.000	H2S	-3.817	-0.000	-0.000	OCS	-22.744	-0.000	0.000
	0.000	-1.171	0.000		-0.000	-5.975	-0.000		-0.000	-22.744	-0.000
	0.000	0.000	-0.649		-0.000	-0.000	-5.393		0.000	-0.000	-5.785
CH2O	-8.468	0.000	0.000	H4C2O	-13.262	-0.000	-0.000	OF2	-0.177	0.000	0.000
	0.000	-5.307	0.000		-0.000	-9.066	0.000		0.000	-2.971	-0.000
	0.000	0.000	-3.220		-0.000	0.000	-10.558		0.000	-0.000	-4.624
CH3F	-3.986	0.000	-0.000	HCN	-7.034	-0.000	0.000	PN	-11.331	-0.000	-0.000
	0.000	-7.085	-0.000		-0.000	-7.034	-0.000		-0.000	-11.331	-0.000
	-0.000	-0.000	-7.085		0.000	-0.000	-3.777		-0.000	-0.000	-6.104
CH4	-4.417	0.000	0.000	HCP	-15.529	-0.000	-0.000	SO2	-17.598	-0.000	-0.000
	0.000	-4.417	-0.000		-0.000	-15.529	0.000		-0.000	-6.306	-0.000
	0.000	-0.000	-4.417		-0.000	0.000	-8.013		-0.000	-0.000	-15.026
CO	-4.933	-0.000	-0.000	HF	-1.143	0.000	-0.000	SiH+	-4.686	0.000	0.000
	-0.000	-4.933	0.000		0.000	-1.143	0.000		0.000	-4.686	0.000
	-0.000	0.000	-2.911		-0.000	0.000	-1.222		0.000	0.000	-1.534

Table 15: $\mathcal{M}$ computed with CCSD in aug-cc-pCVTZ basis

AlH	-0.000	0.691	0.000	H2S	0.000	-0.510	0.000
	-0.691	-0.000	0.000		0.675	0.000	-0.000
	-0.000	0.000	0.000		0.000	0.000	-0.000
BH	0.000	-0.950	0.000	HF	0.000	-0.109	-0.000
	0.950	0.000	-0.000		0.109	0.000	0.000
	0.000	0.000	-0.000		0.000	-0.000	-0.000
BeH-	0.000	2.802	-0.000	HOF	-0.000	-0.000	-0.094
	-2.802	0.000	-0.000		-0.000	-0.000	-0.499
	0.000	0.000	0.000		0.034	-0.115	-0.000
CH+	-0.000	-1.065	-0.000	LiF	-0.000	-0.708	0.000
	1.065	0.000	-0.000		0.708	-0.000	-0.000
	0.000	-0.000	-0.000		0.000	-0.000	0.000
CO	-0.000	0.063	-0.000	LiH	0.000	-1.235	-0.000
	-0.064	-0.000	0.000		1.235	0.000	-0.000
	-0.000	0.000	-0.000		-0.000	0.000	0.000
FNO	-0.000	-0.001	-0.918	N2	0.000	0.000	0.000
	-0.073	-0.000	2.670		-0.000	0.000	0.000
	0.872	-1.331	0.087		0.000	-0.000	0.000
H2	-0.000	-0.000	-0.000	NH3	0.000	0.000	-0.000
	0.000	-0.000	0.000		-0.000	0.000	0.080
	-0.000	-0.000	-0.000		0.000	-0.080	-0.000
H2O	-0.000	-0.142	0.000	SiH+	0.000	0.294	-0.000
	0.169	0.000	0.000		-0.294	-0.000	0.000
	-0.000	0.000	-0.000		0.000	0.000	-0.000

Table 16: $\mathcal{M}$ computed with MP2 in aug-cc-pCVTZ basis

AlF	0.000	0.723	0.000	FCCH	-0.000	-2.044	-0.000	HFCO	-0.000	-0.000	-0.350
	-0.723	-0.000	-0.000		2.044	0.000	-0.000		-0.000	-0.000	-0.134
	-0.000	-0.000	-0.000		-0.000	0.000	-0.000		-0.324	-0.505	-0.000
AlH	-0.000	0.782	-0.000	FCN	0.000	0.703	-0.000	HOF	-0.000	-0.000	-0.103
	-0.782	-0.000	-0.000		-0.703	0.000	-0.000		-0.000	-0.000	-0.483
	0.000	0.000	0.000		0.000	0.000	-0.000		0.029	-0.132	-0.000
BH	-0.000	-1.124	-0.000	FNO	-0.000	-0.000	-4.685	LiF	-0.000	-0.723	0.000
	1.124	0.000	-0.000		-0.000	-0.000	6.577		0.723	-0.000	-0.000
	-0.000	-0.000	-0.000		4.759	-6.634	-0.000		0.000	0.000	-0.000
BeH-	-0.000	2.872	-0.000	H2	-0.000	-0.000	-0.000	LiH	-0.000	-1.258	-0.000
	-2.872	-0.000	-0.000		-0.000	-0.000	-0.000		1.258	-0.000	-0.000
	-0.000	-0.000	-0.000		-0.000	-0.000	-0.000		-0.000	-0.000	-0.000
C2H4	0.000	0.000	-0.000	H2C2O	-0.000	2.597	0.000	N2	0.000	-0.000	0.000
	-0.000	0.000	0.000		-1.975	0.000	-0.000		-0.000	0.000	0.000
	-0.000	-0.000	-0.000		0.000	-0.000	-0.000		0.000	-0.000	0.000
C3H4	-0.000	-0.004	-0.000	H2O	0.000	-0.150	0.000	N2O	0.000	0.118	-0.000
	0.858	0.000	-0.000		0.176	0.000	-0.000		-0.118	-0.000	-0.000
	0.000	-0.000	-0.000		-0.000	-0.000	-0.000		0.000	0.000	-0.000
C4H4	0.000	-0.000	0.000	H2O2	-0.292	-0.000	-0.000	NH3	-0.000	-0.000	-0.000
	0.000	-0.000	-0.001		0.000	0.258	-0.024		-0.000	0.000	0.087
	-0.000	-0.001	-0.000		0.000	0.158	-0.023		0.000	-0.087	-0.000
CH+	-0.000	-1.302	-0.000	H2S	0.000	-0.527	0.000	OCS	0.000	1.975	0.000
	1.302	0.000	-0.000		0.683	0.000	-0.000		-1.975	-0.000	0.000
	-0.000	0.000	-0.000		-0.000	-0.000	0.000		-0.000	-0.000	0.000
CH2O	-0.000	0.478	-0.000	H4C2O	0.000	0.775	-0.000	OF2	0.000	0.171	-0.000
	0.380	-0.000	-0.000		0.483	-0.000	0.000		-0.480	-0.000	-0.000
	0.000	-0.000	0.000		-0.000	-0.000	0.000		-0.000	-0.000	-0.000
CH3F	0.000	-0.000	0.000	HCN	-0.000	-0.351	-0.000	PN	-0.000	-0.927	-0.000
	-0.000	0.000	0.185		0.351	-0.000	0.000		0.927	0.000	0.000
	-0.000	-0.185	0.000		-0.000	0.000	0.000		-0.000	0.000	0.000
CH4	-0.000	0.000	-0.000	HCP	-0.000	-0.028	0.000	SO2	-0.000	-1.055	0.000
	0.000	-0.000	0.000		0.028	0.000	-0.000		-1.208	0.000	-0.000
	-0.000	-0.000	-0.000		0.000	0.000	-0.000		0.000	-0.000	0.000
CO	-0.000	-0.022	0.000	HF	0.000	-0.112	0.000	SiH+	-0.000	0.372	-0.000
	0.022	-0.000	0.000		0.112	-0.000	-0.000		-0.372	0.000	-0.000
	-0.000	0.000	0.000		0.000	0.000	-0.000		0.000	-0.000	-0.000

Table 17: $\mathcal{M}$ computed with HF in aug-cc-pCVTZ basis

AlF	0.000	0.860	0.000	FCCH	-0.000	-2.088	-0.000	HFCO	-0.000	-0.000	-0.265
	-0.860	-0.000	-0.000		2.088	0.000	-0.000		-0.000	-0.000	-0.102
	-0.000	-0.000	-0.000		0.000	0.000	-0.000		-0.302	-0.585	-0.000
AlH	-0.000	0.808	0.000	FCN	0.000	0.743	-0.000	HOF	-0.000	-0.000	-0.046
	-0.808	-0.000	-0.000		-0.743	0.000	-0.000		-0.000	-0.000	-0.740
	0.000	0.000	0.000		0.000	0.000	-0.000		0.047	0.057	-0.000
BH	0.000	-1.328	-0.000	FNO	-0.000	-0.000	1.586	LiF	-0.000	-0.691	0.000
	1.328	0.000	-0.000		-0.000	-0.000	-2.247		0.691	-0.000	-0.000
	0.000	-0.000	-0.000		-1.645	2.283	-0.000		0.000	0.000	-0.000
BeH-	-0.000	2.726	0.000	H2	0.000	-0.000	0.000	LiH	0.000	-1.293	0.000
	-2.726	0.000	0.000		-0.000	0.000	-0.000		1.293	0.000	-0.000
	0.000	0.000	0.000		-0.000	-0.000	0.000		-0.000	-0.000	0.000
C2H4	-0.000	0.000	-0.000	H2C2O	-0.000	2.447	0.000	N2	-0.000	-0.000	0.000
	-0.000	-0.000	0.000		-1.843	0.000	-0.000		-0.000	0.000	0.000
	-0.000	0.000	-0.000		0.000	-0.000	0.000		0.000	-0.000	-0.000
C3H4	-0.000	0.051	-0.000	H2O	0.000	-0.119	0.000	N2O	0.000	0.066	-0.000
	0.707	0.000	-0.000		0.143	0.000	0.000		-0.066	-0.000	-0.000
	0.000	-0.000	-0.000		0.000	-0.000	0.000		0.000	0.000	-0.000
C4H4	-0.000	0.000	0.000	H2O2	-0.270	-0.000	-0.000	NH3	-0.000	-0.000	-0.000
	0.000	-0.000	-0.001		0.000	0.230	-0.045		-0.000	0.000	0.061
	-0.000	-0.001	-0.000		0.000	0.095	-0.013		0.000	-0.061	-0.000
CH+	-0.000	-1.636	0.000	H2S	0.000	-0.470	-0.000	OCS	0.000	1.979	0.000
	1.636	0.000	-0.000		0.625	0.000	-0.000		-1.979	-0.000	0.000
	0.000	0.000	0.000		0.000	-0.000	0.000		-0.000	-0.000	0.000
CH2O	0.000	0.352	-0.000	H4C2O	0.000	0.609	-0.000	OF2	0.000	0.184	-0.000
	0.296	-0.000	-0.000		0.347	-0.000	0.000		-0.367	-0.000	-0.000
	0.000	-0.000	0.000		-0.000	-0.000	0.000		-0.000	-0.000	-0.000
CH3F	0.000	-0.000	0.000	HCN	-0.000	-0.335	-0.000	PN	-0.000	-1.063	-0.000
	-0.000	0.000	0.224		0.335	0.000	0.000		1.063	0.000	0.000
	-0.000	-0.224	0.000		-0.000	0.000	0.000		-0.000	0.000	0.000
CH4	-0.000	0.000	-0.000	HCP	0.000	-0.060	-0.000	SO2	0.000	-1.058	0.000
	0.000	-0.000	0.000		0.060	-0.000	-0.000		-1.191	-0.000	-0.000
	-0.000	-0.000	-0.000		-0.000	-0.000	-0.000		0.000	-0.000	0.000
CO	-0.000	0.205	0.000	HF	0.000	-0.092	0.000	SiH+	-0.000	0.395	-0.000
	-0.205	-0.000	0.000		0.092	0.000	-0.000		-0.395	0.000	-0.000
	-0.000	0.000	0.000		0.000	0.000	-0.000		0.000	-0.000	-0.000

Table 18: $\mathcal{M}$ computed with LDA in aug-cc-pCVTZ basis

AlF	0.000	0.692	0.000	FCCH	-0.000	-2.023	-0.000	HFCO	0.000	-0.000	-0.415
	-0.692	0.000	0.000		2.023	0.000	0.000		-0.000	-0.000	-0.163
	-0.000	-0.000	0.000		-0.000	0.000	-0.000		-0.316	-0.469	0.000
AlH	-0.000	0.676	0.000	FCN	-0.000	0.605	-0.000	HOF	-0.000	-0.000	-0.100
	-0.676	0.000	-0.000		-0.605	-0.000	0.000		0.000	0.000	-0.641
	0.000	0.000	0.000		-0.000	-0.000	0.000		0.018	-0.097	-0.000
BH	0.000	-1.368	0.000	FNO	x	x	x	LiF	-0.000	-0.739	0.000
	1.368	0.000	0.000		x	x	x		0.739	0.000	0.000
	-0.000	-0.000	0.000		x	x	x		-0.000	0.000	-0.000
BeH-	-0.000	3.400	-0.000	H2	0.000	-0.000	-0.000	LiH	-0.000	-1.204	0.000
	-3.400	-0.000	-0.000		0.000	-0.000	0.000		1.204	0.000	0.000
	0.000	-0.000	0.000		0.000	-0.000	-0.000		-0.000	-0.000	-0.000
C2H4	-0.000	0.000	-0.000	H2C2O	0.000	2.682	0.000	N2	-0.000	0.000	0.000
	-0.000	0.000	0.000		-1.897	-0.000	-0.000		-0.000	-0.000	-0.000
	0.000	-0.000	-0.000		-0.000	0.000	-0.000		0.000	-0.000	0.000
C3H4	0.000	0.040	0.000	H2O	0.000	-0.157	-0.000	N2O	0.000	0.105	0.000
	1.000	-0.000	-0.000		0.184	-0.000	0.000		-0.105	0.000	0.000
	0.000	0.000	0.000		0.000	0.000	-0.000		0.000	0.000	-0.000
C4H4	-0.000	0.000	-0.000	H2O2	-0.336	-0.000	-0.000	NH3	-0.000	0.000	-0.000
	0.000	-0.000	0.000		0.000	0.290	0.008		-0.000	0.000	0.091
	-0.000	0.000	-0.000		-0.000	0.163	-0.029		0.000	-0.091	-0.000
CH+	0.000	-1.573	-0.000	H2S	0.000	-0.546	0.000	OCS	-0.000	1.951	-0.000
	1.573	-0.000	0.000		0.710	-0.000	0.000		-1.951	-0.000	-0.000
	-0.000	0.000	-0.000		-0.000	0.000	-0.000		0.000	0.000	-0.000
CH2O	-0.000	0.504	-0.000	H4C2O	-0.000	0.851	0.000	OF2	0.000	0.216	-0.000
	0.423	0.000	0.000		0.526	-0.000	0.000		-0.906	-0.000	-0.000
	0.000	0.000	-0.000		0.000	0.000	-0.000		0.000	-0.000	0.000
CH3F	0.000	-0.000	0.000	HCN	-0.000	-0.414	0.000	PN	0.000	-1.118	0.000
	0.000	0.000	0.179		0.414	-0.000	0.000		1.118	-0.000	-0.000
	0.000	-0.179	-0.000		0.000	0.000	-0.000		0.000	0.000	-0.000
CH4	0.000	0.000	0.000	HCP	-0.000	-0.012	0.000	SO2	0.000	-1.045	0.000
	0.000	-0.000	-0.000		0.012	0.000	0.000		-1.439	-0.000	0.000
	-0.000	-0.000	0.000		-0.000	-0.000	0.000		-0.000	0.000	0.000
CO	-0.000	0.050	-0.000	HF	-0.000	-0.120	-0.000	SiH+	-0.000	0.322	0.000
	-0.050	0.000	0.000		0.120	0.000	-0.000		-0.322	0.000	0.000
	-0.000	0.000	0.000		-0.000	-0.000	-0.000		0.000	-0.000	0.000

Table 19: $\mathcal{M}$ computed with KT3 in aug-cc-pCVTZ basis

AlF	-0.000	0.725	0.000	FCCH	-0.000	-2.107	-0.000	HFCO	-0.000	-0.000	-0.394
	-0.725	-0.000	-0.000		2.107	0.000	-0.000		0.000	-0.000	-0.098
	-0.000	0.000	0.000		0.000	-0.000	0.000		-0.315	-0.462	-0.000
AlH	-0.000	0.762	0.000	FCN	0.000	0.730	-0.000	HOF	0.000	0.000	-0.159
	-0.762	0.000	-0.000		-0.730	-0.000	-0.000		-0.000	0.000	-0.537
	0.000	0.000	-0.000		0.000	0.000	-0.000		0.029	-0.116	0.000
BH	0.000	-0.825	0.000	FNO	0.000	-0.000	-2.517	LiF	0.000	-0.731	0.000
	0.825	0.000	-0.000		-0.000	-0.000	3.530		0.731	0.000	0.000
	0.000	-0.000	0.000		2.637	-3.608	0.000		0.000	-0.000	0.000
BeH-	0.000	3.066	0.000	H2	-0.000	-0.000	-0.000	LiH	-0.000	-1.122	0.000
	-3.066	0.000	-0.000		0.000	-0.000	0.000		1.122	-0.000	0.000
	0.000	-0.000	-0.000		-0.000	-0.000	-0.000		-0.000	0.000	0.000
C2H4	0.000	-0.000	-0.000	H2C2O	0.000	2.505	0.000	N2	0.000	-0.000	0.000
	0.000	-0.000	0.000		-1.888	0.000	0.000		-0.000	0.000	0.000
	0.000	-0.000	0.000		-0.000	0.000	0.000		-0.000	0.000	-0.000
C3H4	-0.000	0.051	-0.000	H2O	-0.000	-0.185	-0.000	N2O	-0.000	0.156	-0.000
	0.881	0.000	-0.000		0.225	0.000	-0.000		-0.156	0.000	0.000
	-0.000	-0.000	-0.000		-0.000	0.000	0.000		0.000	-0.000	-0.000
C4H4	0.000	-0.000	0.000	H2O2	-0.327	-0.000	-0.000	NH3	0.000	-0.000	-0.000
	0.000	0.000	0.000		-0.000	0.286	-0.014		0.000	-0.000	0.138
	0.000	-0.000	0.000		0.000	0.215	-0.034		0.000	-0.138	-0.000
CH+	-0.000	-0.925	-0.000	H2S	-0.000	-0.524	0.000	OCS	-0.000	1.951	-0.000
	0.925	-0.000	0.000		0.714	-0.000	-0.000		-1.951	-0.000	-0.000
	0.000	0.000	-0.000		-0.000	0.000	-0.000		0.000	-0.000	0.000
CH2O	-0.000	0.470	-0.000	H4C2O	-0.000	0.814	-0.000	OF2	0.000	0.235	-0.000
	0.370	0.000	0.000		0.467	-0.000	0.000		-0.664	-0.000	0.000
	-0.000	0.000	0.000		0.000	0.000	0.000		0.000	0.000	-0.000
CH3F	0.000	0.000	0.000	HCN	-0.000	-0.361	0.000	PN	0.000	-1.107	0.000
	0.000	0.000	0.175		0.361	-0.000	0.000		1.107	-0.000	-0.000
	0.000	-0.175	0.000		-0.000	0.000	-0.000		0.000	0.000	0.000
CH4	0.000	0.000	0.000	HCP	-0.000	0.075	0.000	SO2	0.000	-1.010	-0.000
	0.000	0.000	0.000		-0.075	-0.000	-0.000		-1.437	0.000	0.000
	-0.000	0.000	-0.000		0.000	0.000	-0.000		-0.000	0.000	-0.000
CO	0.000	0.037	-0.000	HF	0.000	-0.138	0.000	SiH+	-0.000	0.284	0.000
	-0.037	0.000	-0.000		0.138	0.000	0.000		-0.284	0.000	-0.000
	-0.000	0.000	0.000		-0.000	0.000	-0.000		-0.000	-0.000	0.000

Table 20: $\mathcal{M}$ computed with M06 in aug-cc-pCVTZ basis

AlF	0.000	0.887	-0.000	FCCH	0.000	-2.441	0.000	HFCO	-0.000	0.000	-0.473
	-0.887	-0.000	-0.000		2.441	0.000	0.000		-0.000	0.000	-0.175
	0.000	-0.000	-0.000		0.000	-0.000	0.000		-0.448	-0.386	-0.000
AlH	0.000	0.905	-0.000	FCN	0.000	0.856	-0.000	HOF	0.000	-0.000	-0.126
	-0.905	0.000	0.000		-0.856	-0.000	0.000		0.000	0.000	-0.616
	0.000	-0.000	0.000		-0.000	0.000	-0.000		-0.027	-0.144	0.000
BH	0.000	-1.062	-0.000	FNO	x	x	x	LiF	-0.000	-0.997	0.000
	1.062	0.000	0.000		x	x	x		0.997	0.000	-0.000
	-0.000	-0.000	0.000		x	x	x		0.000	0.000	0.000
BeH-	x	x	x	H2	-0.000	0.000	0.000	LiH	0.000	-1.039	0.000
	x	x	x		-0.000	-0.000	-0.000		1.039	0.000	0.000
	x	x	x		-0.000	-0.000	0.000		0.000	0.000	-0.000
C2H4	-0.000	0.000	-0.000	H2C2O	-0.000	3.000	-0.000	N2	-0.000	-0.000	-0.000
	-0.000	0.000	0.000		-2.353	0.000	0.000		0.000	0.000	0.000
	0.000	0.000	-0.000		-0.000	-0.000	0.000		0.000	-0.000	0.000
C3H4	-0.000	0.090	0.000	H2O	-0.000	-0.197	0.000	N2O	-0.000	0.216	0.000
	0.990	0.000	-0.000		0.229	0.000	0.000		-0.216	0.000	-0.000
	0.000	-0.000	0.000		-0.000	0.000	-0.000		-0.000	-0.000	-0.000
C4H4	-0.000	0.000	0.000	H2O2	-0.359	0.000	-0.000	NH3	0.000	-0.000	-0.000
	0.000	0.000	0.000		0.000	0.268	0.084		-0.000	0.000	0.119
	-0.000	0.000	-0.000		-0.000	0.218	-0.038		0.000	-0.119	-0.000
CH+	0.000	-1.350	-0.000	H2S	0.000	-0.594	0.000	OCS	0.000	2.472	-0.000
	1.350	0.000	0.000		0.786	-0.000	-0.000		-2.472	0.000	-0.000
	-0.000	0.000	0.000		0.000	-0.000	-0.000		-0.000	-0.000	-0.000
CH2O	-0.000	0.809	-0.000	H4C2O	0.000	1.183	-0.000	OF2	0.000	0.313	0.000
	0.465	0.000	0.000		0.858	-0.000	0.000		-0.922	-0.000	0.000
	0.000	0.000	0.000		0.000	0.000	0.000		-0.000	0.000	0.000
CH3F	-0.000	-0.000	-0.000	HCN	-0.000	-0.415	0.000	PN	-0.000	-1.189	-0.000
	-0.000	-0.000	0.097		0.415	0.000	-0.000		1.189	-0.000	0.000
	0.000	-0.097	0.000		0.000	0.000	-0.000		-0.000	-0.000	0.000
CH4	-0.000	0.000	-0.000	HCP	0.000	0.059	-0.000	SO2	-0.000	-1.209	0.000
	0.000	-0.000	0.000		-0.059	-0.000	0.000		-1.449	-0.000	0.000
	-0.000	0.000	0.000		0.000	0.000	-0.000		-0.000	0.000	-0.000
CO	-0.000	-0.088	-0.000	HF	-0.000	-0.153	-0.000	SiH+	0.000	0.370	0.000
	0.088	0.000	-0.000		0.153	-0.000	0.000		-0.370	-0.000	0.000
	-0.000	0.000	-0.000		0.000	-0.000	-0.000		-0.000	-0.000	0.000

Table 21: $\mathcal{M}$ computed with TPSS in aug-cc-pCVTZ basis

AlF	0.000	0.744	-0.000	FCCH	0.000	-2.022	-0.000	HFCO	-0.000	-0.000	-0.401
	-0.744	-0.000	0.000		2.022	0.000	-0.000		0.000	-0.000	-0.141
	0.000	-0.000	0.000		0.000	0.000	0.000		-0.307	-0.476	-0.000
AlH	0.000	0.706	0.000	FCN	0.000	0.652	-0.000	HOF	-0.000	-0.000	-0.126
	-0.706	0.000	-0.000		-0.652	-0.000	-0.000		0.000	-0.000	-0.559
	0.000	0.000	-0.000		0.000	0.000	0.000		0.026	-0.105	-0.000
BH	-0.000	-0.950	-0.000	FNO	0.000	0.000	-4.215	LiF	-0.000	-0.733	-0.000
	0.950	-0.000	0.000		0.000	-0.000	5.929		0.733	0.000	-0.000
	0.000	-0.000	-0.000		5.446	-6.575	-0.000		-0.000	0.000	0.000
BeH-	-0.000	3.407	-0.000	H2	-0.000	0.000	0.000	LiH	0.000	-1.178	0.000
	-3.407	0.000	-0.000		-0.000	0.000	-0.000		1.178	-0.000	-0.000
	-0.000	-0.000	0.000		-0.000	0.000	0.000		-0.000	-0.000	-0.000
C2H4	0.000	0.000	-0.000	H2C2O	-0.000	2.545	-0.000	N2	0.000	-0.000	-0.000
	0.000	0.000	-0.000		-1.847	-0.000	0.000		0.000	-0.000	-0.000
	0.000	-0.000	-0.000		-0.000	-0.000	-0.000		0.000	0.000	-0.000
C3H4	0.000	0.049	-0.000	H2O	-0.000	-0.168	0.000	N2O	0.000	0.124	0.000
	0.888	-0.000	0.000		0.201	-0.000	0.000		-0.124	-0.000	0.000
	0.000	-0.000	-0.000		-0.000	-0.000	-0.000		0.000	0.000	-0.000
C4H4	-0.000	0.000	-0.000	H2O2	-0.319	-0.000	-0.000	NH3	0.000	-0.000	-0.000
	0.000	0.000	0.000		0.000	0.277	-0.011		-0.000	-0.000	0.111
	-0.000	-0.000	0.000		0.000	0.183	-0.029		0.000	-0.111	0.000
CH+	0.000	-1.070	0.000	H2S	0.000	-0.511	-0.000	OCS	-0.000	1.960	-0.000
	1.070	-0.000	-0.000		0.689	-0.000	0.000		-1.960	0.000	-0.000
	-0.000	0.000	-0.000		0.000	0.000	0.000		0.000	-0.000	-0.000
CH2O	-0.000	0.451	-0.000	H4C2O	-0.000	0.786	-0.000	OF2	-0.000	0.229	-0.000
	0.387	0.000	0.000		0.465	0.000	-0.000		-0.697	-0.000	0.000
	0.000	-0.000	-0.000		0.000	0.000	0.000		0.000	0.000	-0.000
CH3F	0.000	-0.000	0.000	HCN	0.000	-0.375	0.000	PN	0.000	-1.177	-0.000
	-0.000	-0.000	0.175		0.375	0.000	0.000		1.177	0.000	-0.000
	-0.000	-0.175	-0.000		-0.000	0.000	-0.000		0.000	0.000	0.000
CH4	-0.000	0.000	-0.000	HCP	-0.000	0.084	0.000	SO2	0.000	-1.024	-0.000
	0.000	0.000	0.000		-0.084	-0.000	-0.000		-1.463	-0.000	-0.000
	-0.000	-0.000	0.000		0.000	0.000	-0.000		-0.000	-0.000	0.000
CO	0.000	0.052	-0.000	HF	0.000	-0.128	-0.000	SiH+	0.000	0.257	0.000
	-0.052	-0.000	-0.000		0.128	0.000	-0.000		-0.257	0.000	-0.000
	0.000	0.000	-0.000		0.000	0.000	-0.000		-0.000	0.000	-0.000

Table 22: Eigenvalues of ζ, α_ζ, computed with CCSD in aug-cc-pCVTZ basis. The average eigenvalue is, $\bar{\alpha}_\zeta = \frac{1}{6}\mathrm{Tr}(\zeta)$

Mol	eig.avg	eig.1	eig.2	eig.3	eig.4	eig.5	eig.6
AlH	-1.793	2.956	2.956	-2.026	-4.565	-5.038	-5.039
BH	0.426	5.245	5.244	-0.921	-2.263	-2.264	-2.485
BeH-	-15.627	3.042	3.042	-7.289	-21.028	-35.763	-35.763
CH+	1.765	6.909	6.909	-0.319	-0.733	-0.733	-1.441
CO	-2.384	-1.428	-2.088	-2.091	-2.439	-2.441	-3.815
FNO	-0.713	4.585	3.980	-2.058	-2.347	-3.049	-5.390
H2	-0.609	-0.376	-0.391	-0.391	-0.763	-0.867	-0.867
H2O	-1.931	-0.724	-0.926	-0.982	-2.964	-2.988	-3.003
H2S	-4.149	-1.827	-2.665	-2.842	-5.597	-5.685	-6.274
HF	-1.385	-0.518	-0.518	-0.556	-2.163	-2.278	-2.278
HOF	-1.845	0.185	-0.929	-1.125	-2.293	-2.573	-4.334
LiF	-1.787	-0.853	-0.879	-0.879	-2.699	-2.699	-2.717
LiH	-2.187	-0.818	-0.818	-1.769	-1.992	-3.862	-3.863
N2	-2.328	-1.395	-2.020	-2.020	-2.357	-2.357	-3.816
NH3	-2.555	-1.146	-1.574	-1.574	-3.537	-3.751	-3.751
SiH+	-0.457	2.964	2.964	-0.771	-2.377	-2.377	-3.142

Table 23: Eigenvalues of ζ, α_ζ, computed with MP2 in aug-cc-pCVTZ basis. The average eigenvalue is, $\bar{\alpha}_\zeta = \frac{1}{6}\text{Tr}(\zeta)$

Mol	eig.avg	eig.1	eig.2	eig.3	eig.4	eig.5	eig.6
AlF	-5.741	-3.390	-4.561	-4.561	-5.903	-8.016	-8.016
AlH	-1.703	3.449	3.449	-2.045	-4.567	-5.251	-5.251
BH	0.783	6.435	6.435	-0.942	-2.365	-2.365	-2.502
BeH-	-17.131	3.869	3.869	-7.505	-23.225	-39.897	-39.897
C2H4	-4.979	-3.380	-3.570	-4.136	-5.617	-6.062	-7.110
C3H4	-6.699	-3.973	-5.221	-5.734	-7.411	-8.643	-9.213
C4H4	-7.343	0.213	-4.397	-5.456	-8.683	-12.203	-13.532
CH+	2.332	8.622	8.622	-0.321	-0.745	-0.745	-1.443
CH2O	-2.271	0.048	-1.642	-1.875	-2.397	-3.418	-4.339
CH3F	-3.566	-2.025	-3.321	-3.321	-3.752	-3.752	-5.222
CH4	-3.155	-2.235	-2.235	-2.235	-4.075	-4.075	-4.076
CO	-2.459	-1.483	-2.184	-2.184	-2.528	-2.528	-3.844
FCCH	-6.331	-2.398	-4.447	-4.447	-6.351	-10.171	-10.171
FCN	-4.787	-1.635	-4.071	-4.071	-5.665	-6.642	-6.642
FNO	-0.315	10.822	10.806	-1.398	-5.753	-8.131	-8.235
H2	-0.613	-0.380	-0.394	-0.394	-0.768	-0.870	-0.870
H2C2O	-6.774	-2.767	-4.127	-4.738	-6.009	-11.440	-11.563
H2O	-1.974	-0.744	-0.966	-1.037	-3.010	-3.035	-3.052
H2O2	-2.604	-0.971	-1.287	-1.957	-3.038	-3.151	-5.219
H2S	-4.228	-1.842	-2.730	-2.921	-5.731	-5.792	-6.348
H4C2O	-6.260	-4.477	-5.354	-5.389	-6.899	-7.086	-8.357
HCN	-3.257	-1.898	-2.886	-2.886	-3.747	-3.747	-4.376
HCP	-6.546	-4.123	-5.839	-5.839	-7.434	-8.019	-8.019
HF	-1.411	-0.540	-0.540	-0.584	-2.189	-2.307	-2.307
HFCO	-4.444	-2.258	-3.546	-3.597	-4.503	-6.000	-6.759
HOF	-1.857	0.234	-0.951	-1.136	-2.311	-2.589	-4.388
LiF	-1.832	-0.912	-0.914	-0.914	-2.740	-2.740	-2.769
LiH	-2.153	-0.771	-0.771	-1.715	-1.961	-3.848	-3.848
N2	-2.371	-1.411	-2.098	-2.098	-2.404	-2.404	-3.813
N2O	-4.478	-1.280	-3.679	-3.679	-5.720	-6.256	-6.256
NH3	-2.610	-1.160	-1.639	-1.639	-3.598	-3.811	-3.811
OCS	-8.155	-2.998	-6.186	-6.186	-8.739	-12.411	-12.411
OF2	-2.325	-0.449	-1.413	-2.172	-2.431	-3.372	-4.113
PN	-4.588	-2.669	-2.669	-3.176	-6.211	-6.211	-6.589
SO2	-5.483	-2.746	-3.578	-3.916	-5.351	-7.859	-9.449
SiH+	-0.348	3.348	3.348	-0.769	-2.436	-2.436	-3.142

Table 24: Eigenvalues of ζ, α_ζ, computed with HF in aug-cc-pCVTZ basis. The average eigenvalue is, $\bar{\alpha}_\zeta = \frac{1}{6}\mathrm{Tr}(\zeta)$

Mol	eig.avg	eig.1	eig.2	eig.3	eig.4	eig.5	eig.6
AlF	-5.774	-3.365	-4.480	-4.480	-5.837	-8.240	-8.240
AlH	-1.833	3.556	3.556	-2.244	-4.632	-5.617	-5.617
BH	1.030	7.227	7.227	-0.963	-2.400	-2.400	-2.511
BeH-	-16.872	4.157	4.157	-7.601	-23.235	-39.354	-39.354
C2H4	-4.993	-3.444	-3.545	-4.277	-5.666	-6.011	-7.016
C3H4	-6.642	-4.062	-5.225	-5.403	-7.525	-8.627	-9.011
C4H4	-7.280	0.435	-4.419	-5.078	-8.473	-12.537	-13.607
CH+	3.020	10.691	10.691	-0.324	-0.747	-0.747	-1.443
CH2O	-2.316	-0.112	-1.680	-1.745	-2.650	-3.578	-4.129
CH3F	-3.548	-1.993	-3.315	-3.315	-3.776	-3.776	-5.115
CH4	-3.076	-2.175	-2.175	-2.175	-3.977	-3.977	-3.977
CO	-2.309	-1.396	-1.896	-1.896	-2.433	-2.433	-3.801
FCCH	-6.352	-2.447	-4.482	-4.482	-6.398	-10.150	-10.150
FCN	-4.789	-1.682	-4.102	-4.102	-5.681	-6.584	-6.584
FNO	4.925	19.887	19.799	-1.311	-1.619	-1.645	-5.560
H2	-0.620	-0.388	-0.400	-0.400	-0.777	-0.876	-0.876
H2C2O	-6.716	-2.740	-4.160	-4.924	-5.815	-11.283	-11.372
H2O	-1.882	-0.713	-0.872	-0.897	-2.910	-2.938	-2.961
H2O2	-2.629	-1.029	-1.132	-1.911	-3.289	-3.373	-5.042
H2S	-4.130	-1.840	-2.672	-2.846	-5.502	-5.630	-6.288
H4C2O	-6.281	-4.396	-5.441	-5.587	-6.954	-7.109	-8.196
HCN	-3.263	-1.966	-2.905	-2.905	-3.676	-3.676	-4.453
HCP	-6.637	-4.352	-5.928	-5.928	-7.605	-8.003	-8.003
HF	-1.340	-0.481	-0.481	-0.503	-2.115	-2.230	-2.230
HFCO	-4.357	-2.161	-3.404	-3.621	-4.472	-5.973	-6.509
HOF	-1.835	0.388	-0.829	-1.066	-2.529	-2.730	-4.244
LiF	-1.713	-0.746	-0.819	-0.819	-2.614	-2.641	-2.641
LiH	-2.143	-0.732	-0.732	-1.681	-1.950	-3.882	-3.882
N2	-2.299	-1.429	-1.924	-1.924	-2.328	-2.328	-3.863
N2O	-4.435	-1.293	-3.647	-3.647	-5.732	-6.146	-6.146
NH3	-2.515	-1.167	-1.495	-1.495	-3.506	-3.715	-3.715
OCS	-8.246	-3.044	-6.258	-6.258	-8.807	-12.555	-12.555
OF2	-2.438	-0.689	-1.186	-2.366	-2.481	-3.575	-4.333
PN	-4.363	-2.129	-2.129	-3.250	-6.004	-6.004	-6.660
SO2	-5.196	-2.546	-2.617	-3.767	-5.383	-7.674	-9.189
SiH+	-0.351	3.437	3.437	-0.790	-2.517	-2.517	-3.154

Table 25: Eigenvalues of ζ, α_ζ, computed with LDA in aug-cc-pCVTZ basis. The average eigenvalue is, $\bar{\alpha}_\zeta = \frac{1}{6}\mathrm{Tr}(\zeta)$

Mol	eig.avg	eig.1	eig.2	eig.3	eig.4	eig.5	eig.6
AlF	-5.676	-3.428	-4.423	-4.423	-5.952	-7.915	-7.915
AlH	-1.387	4.507	4.507	-1.985	-4.632	-5.360	-5.360
BH	1.131	7.698	7.698	-1.017	-2.507	-2.507	-2.579
BeH-	-28.706	4.792	4.792	-8.638	-38.807	-67.188	-67.188
C2H4	-4.864	-3.299	-3.331	-3.751	-5.542	-6.080	-7.177
C3H4	-6.525	-3.659	-5.178	-5.375	-7.366	-8.451	-9.119
C4H4	-6.952	0.903	-4.006	-4.852	-8.649	-12.034	-13.073
CH+	2.909	10.381	10.381	-0.337	-0.751	-0.751	-1.471
CH2O	-2.047	0.977	-1.601	-1.624	-2.291	-3.344	-4.399
CH3F	-3.541	-2.041	-3.258	-3.258	-3.693	-3.693	-5.300
CH4	-3.239	-2.301	-2.301	-2.301	-4.178	-4.178	-4.178
CO	-2.399	-1.501	-1.993	-1.993	-2.520	-2.520	-3.868
FCCH	-6.278	-2.439	-4.319	-4.319	-6.397	-10.097	-10.097
FCN	-4.680	-1.641	-3.938	-3.938	-5.710	-6.426	-6.426
H2	-0.665	-0.439	-0.455	-0.455	-0.807	-0.916	-0.916
H2C2O	-6.673	-2.739	-3.951	-4.646	-5.955	-11.198	-11.548
H2O	-2.004	-0.740	-0.999	-1.086	-3.043	-3.067	-3.086
H2O2	-2.425	-0.557	-1.251	-1.764	-2.799	-2.942	-5.235
H2S	-4.248	-1.780	-2.765	-2.972	-5.777	-5.822	-6.372
H4C2O	-6.146	-4.441	-5.154	-5.382	-6.729	-6.887	-8.287
HCN	-3.197	-1.926	-2.652	-2.652	-3.769	-3.769	-4.414
HCP	-6.358	-4.075	-5.373	-5.373	-7.426	-7.951	-7.951
HF	-1.446	-0.571	-0.571	-0.625	-2.226	-2.343	-2.343
HFCO	-4.334	-2.285	-3.217	-3.351	-4.476	-5.944	-6.731
HOF	-1.662	0.795	-0.784	-1.111	-2.079	-2.373	-4.418
LiF	-1.846	-0.911	-0.911	-1.004	-2.705	-2.705	-2.841
LiH	-2.400	-0.962	-0.962	-2.063	-2.136	-4.138	-4.138
N2	-2.322	-1.443	-1.896	-1.896	-2.422	-2.422	-3.854
N2O	-4.380	-1.275	-3.491	-3.491	-5.724	-6.150	-6.150
NH3	-2.649	-1.139	-1.704	-1.704	-3.642	-3.853	-3.853
OCS	-7.944	-2.922	-5.929	-5.929	-8.686	-12.097	-12.097
OF2	-1.942	0.711	-1.407	-1.931	-2.280	-2.942	-3.806
PN	-4.252	-1.815	-1.815	-3.109	-6.097	-6.097	-6.577
SO2	-5.173	-2.667	-2.766	-3.483	-5.218	-7.617	-9.286
SiH+	-0.030	4.261	4.261	-0.750	-2.397	-2.397	-3.161

Table 26: Eigenvalues of ζ, α_ζ, computed with KT3 in aug-cc-pCVTZ basis. The average eigenvalue is, $\bar{\alpha}_\zeta = \frac{1}{6}\text{Tr}(\zeta)$

Mol	eig.avg	eig.1	eig.2	eig.3	eig.4	eig.5	eig.6
AlF	-5.693	-3.495	-4.413	-4.413	-5.880	-7.979	-7.979
AlH	-1.811	3.283	3.283	-2.064	-4.578	-5.397	-5.397
BH	0.420	5.476	5.476	-0.911	-2.502	-2.510	-2.510
BeH-	-29.712	5.116	5.116	-8.426	-34.628	-72.726	-72.726
C2H4	-4.821	-3.335	-3.409	-3.873	-5.360	-5.918	-7.032
C3H4	-6.314	-3.626	-4.915	-5.333	-7.083	-8.134	-8.794
C4H4	-6.882	1.024	-4.083	-5.067	-8.301	-11.888	-12.974
CH+	1.590	6.398	6.398	-0.313	-0.755	-0.755	-1.435
CH2O	-2.153	0.201	-1.606	-1.675	-2.258	-3.277	-4.303
CH3F	-3.428	-1.995	-3.174	-3.174	-3.579	-3.579	-5.067
CH4	-3.079	-2.206	-2.206	-2.206	-3.953	-3.953	-3.953
CO	-2.389	-1.486	-2.014	-2.014	-2.508	-2.508	-3.805
FCCH	-6.241	-2.448	-4.248	-4.248	-6.330	-10.088	-10.088
FCN	-4.671	-1.634	-3.896	-3.896	-5.642	-6.480	-6.480
FNO	4.837	19.679	19.546	-1.173	-1.675	-1.769	-5.589
H2	-0.632	-0.414	-0.429	-0.429	-0.768	-0.877	-0.877
H2C2O	-6.524	-2.688	-3.917	-4.572	-5.753	-11.034	-11.182
H2O	-1.948	-0.707	-0.979	-1.062	-2.967	-2.983	-2.989
H2O2	-2.416	-0.742	-1.232	-1.795	-2.753	-2.881	-5.091
H2S	-4.132	-1.732	-2.736	-2.938	-5.546	-5.616	-6.226
H4C2O	-5.955	-4.257	-5.091	-5.178	-6.526	-6.628	-8.051
HCN	-3.197	-1.927	-2.734	-2.734	-3.710	-3.710	-4.365
HCP	-6.369	-4.112	-5.518	-5.518	-7.365	-7.849	-7.849
HF	-1.414	-0.562	-0.562	-0.615	-2.175	-2.284	-2.284
HFCO	-4.298	-2.249	-3.314	-3.380	-4.377	-5.810	-6.660
HOF	-1.717	0.404	-0.894	-1.112	-2.053	-2.340	-4.307
LiF	-1.817	-0.875	-0.875	-1.019	-2.671	-2.671	-2.792
LiH	-2.376	-1.076	-1.076	-2.041	-2.047	-4.008	-4.008
N2	-2.316	-1.442	-1.919	-1.919	-2.405	-2.405	-3.805
N2O	-4.310	-1.237	-3.398	-3.398	-5.651	-6.089	-6.089
NH3	-2.553	-1.069	-1.656	-1.656	-3.490	-3.723	-3.723
OCS	-7.894	-2.906	-5.875	-5.875	-8.599	-12.054	-12.054
OF2	-2.080	-0.067	-1.380	-1.967	-2.289	-2.998	-3.777
PN	-4.286	-1.954	-1.954	-3.134	-6.075	-6.075	-6.521
SO2	-5.101	-2.666	-2.735	-3.442	-5.161	-7.460	-9.139
SiH+	-0.346	3.279	3.279	-0.752	-2.384	-2.384	-3.115

Table 27: Eigenvalues of ζ, α_ζ, computed with M06 in aug-cc-pCVTZ basis. The average eigenvalue is, $\bar{\alpha}_\zeta = \frac{1}{6}\mathrm{Tr}(\zeta)$

Mol	eig.avg	eig.1	eig.2	eig.3	eig.4	eig.5	eig.6
AlF	-6.667	-4.502	-4.835	-4.835	-6.561	-9.634	-9.634
AlH	-1.720	5.603	5.603	-1.915	-5.168	-7.221	-7.221
BH	0.503	7.118	7.118	-1.141	-2.901	-3.587	-3.587
C2H4	-5.434	-3.516	-4.024	-4.060	-5.632	-7.080	-8.294
C3H4	-6.989	-3.418	-5.355	-5.680	-8.403	-8.933	-10.144
C4H4	-7.456	1.573	-3.368	-5.176	-9.329	-14.051	-14.388
CH+	2.363	8.955	8.955	-0.363	-0.892	-0.892	-1.585
CH2O	-2.112	1.252	-1.635	-1.740	-1.757	-3.390	-5.399
CH3F	-3.744	-2.271	-3.204	-3.204	-4.071	-4.071	-5.645
CH4	-3.607	-2.730	-2.730	-2.730	-4.484	-4.484	-4.484
CO	-2.878	-1.977	-2.274	-2.274	-3.258	-3.258	-4.226
FCCH	-7.052	-3.033	-4.375	-4.375	-7.002	-11.763	-11.763
FCN	-5.244	-1.870	-4.053	-4.053	-6.197	-7.645	-7.645
H2	-0.772	-0.563	-0.593	-0.593	-0.881	-1.000	-1.000
H2C2O	-7.390	-3.088	-4.108	-4.478	-6.767	-12.571	-13.324
H2O	-2.263	-0.798	-1.273	-1.443	-3.347	-3.355	-3.359
H2O2	-2.575	-0.563	-1.438	-2.180	-2.692	-2.835	-5.740
H2S	-4.924	-2.031	-3.815	-4.118	-6.302	-6.329	-6.950
H4C2O	-6.526	-4.885	-5.054	-5.797	-6.926	-7.465	-9.029
HCN	-3.644	-2.425	-2.851	-2.851	-4.475	-4.475	-4.790
HCP	-7.288	-5.310	-5.626	-5.626	-8.069	-9.548	-9.548
HF	-1.647	-0.723	-0.723	-0.830	-2.459	-2.573	-2.573
HFCO	-4.901	-2.711	-3.552	-3.612	-4.811	-6.763	-7.954
HOF	-1.840	0.812	-1.152	-1.409	-2.069	-2.375	-4.846
LiF	-2.062	-0.822	-0.822	-1.514	-2.980	-2.980	-3.254
LiH	-3.359	-1.865	-1.865	-2.592	-3.196	-5.317	-5.317
N2	-2.733	-1.895	-2.081	-2.081	-3.078	-3.078	-4.187
N2O	-4.826	-1.288	-3.434	-3.434	-6.191	-7.305	-7.305
NH3	-2.992	-1.148	-2.248	-2.248	-3.940	-4.183	-4.183
OCS	-8.796	-3.555	-5.951	-5.951	-9.436	-13.941	-13.941
OF2	-2.237	0.696	-1.712	-2.350	-3.007	-3.055	-3.996
PN	-4.956	-1.769	-1.769	-4.047	-7.134	-7.507	-7.507
SO2	-6.102	-3.264	-3.509	-3.829	-5.829	-9.122	-11.059
SiH+	-0.058	4.628	4.628	-0.739	-2.751	-2.751	-3.364

Table 28: Eigenvalues of ζ, α_ζ, computed with TPSS in aug-cc-pCVTZ basis. The average eigenvalue is, $\bar{\alpha}_\zeta = \frac{1}{6}\mathrm{Tr}(\zeta)$

Mol	eig.avg	eig.1	eig.2	eig.3	eig.4	eig.5	eig.6
AlF	-5.663	-3.386	-4.398	-4.398	-5.919	-7.938	-7.938
AlH	-1.831	3.052	3.052	-2.082	-4.576	-5.217	-5.217
BH	0.441	5.440	5.440	-0.966	-2.386	-2.386	-2.495
BeH-	-24.066	3.161	3.161	-7.931	-31.060	-55.864	-55.864
C2H4	-4.821	-3.296	-3.361	-3.878	-5.460	-5.919	-7.013
C3H4	-6.425	-3.786	-5.068	-5.318	-7.186	-8.291	-8.904
C4H4	-6.999	0.509	-4.195	-4.941	-8.408	-11.865	-13.091
CH+	1.767	6.919	6.919	-0.325	-0.738	-0.738	-1.437
CH2O	-2.162	0.300	-1.610	-1.636	-2.368	-3.367	-4.291
CH3F	-3.483	-1.993	-3.251	-3.251	-3.647	-3.647	-5.107
CH4	-3.089	-2.208	-2.208	-2.208	-3.970	-3.970	-3.971
CO	-2.359	-1.455	-1.971	-1.971	-2.472	-2.472	-3.813
FCCH	-6.232	-2.402	-4.318	-4.318	-6.341	-10.008	-10.008
FCN	-4.668	-1.624	-3.936	-3.936	-5.660	-6.427	-6.427
FNO	15.946	48.131	48.125	5.710	-0.210	-0.750	-5.331
H2	-0.621	-0.395	-0.410	-0.410	-0.765	-0.872	-0.872
H2C2O	-6.546	-2.684	-3.904	-4.656	-5.773	-11.061	-11.199
H2O	-1.963	-0.732	-0.979	-1.055	-2.991	-3.007	-3.013
H2O2	-2.462	-0.770	-1.238	-1.831	-2.834	-2.960	-5.139
H2S	-4.134	-1.799	-2.696	-2.885	-5.544	-5.634	-6.245
H4C2O	-6.065	-4.360	-5.219	-5.279	-6.640	-6.785	-8.107
HCN	-3.165	-1.888	-2.683	-2.683	-3.686	-3.686	-4.365
HCP	-6.310	-4.006	-5.470	-5.470	-7.376	-7.768	-7.768
HF	-1.425	-0.562	-0.562	-0.611	-2.197	-2.308	-2.308
HFCO	-4.310	-2.244	-3.276	-3.357	-4.446	-5.883	-6.657
HOF	-1.732	0.460	-0.877	-1.108	-2.120	-2.398	-4.350
LiF	-1.829	-0.895	-0.895	-0.994	-2.693	-2.693	-2.806
LiH	-2.219	-0.876	-0.876	-1.843	-1.998	-3.860	-3.860
N2	-2.284	-1.413	-1.863	-1.863	-2.379	-2.379	-3.809
N2O	-4.344	-1.258	-3.464	-3.464	-5.672	-6.104	-6.104
NH3	-2.570	-1.126	-1.646	-1.646	-3.512	-3.746	-3.746
OCS	-7.918	-2.892	-5.915	-5.915	-8.635	-12.076	-12.076
OF2	-2.094	0.040	-1.382	-1.992	-2.312	-3.062	-3.858
PN	-4.198	-1.778	-1.778	-3.052	-6.022	-6.022	-6.534
SO2	-5.094	-2.639	-2.650	-3.421	-5.192	-7.513	-9.147
SiH+	-0.390	3.132	3.132	-0.767	-2.355	-2.355	-3.123

Table 29: Error norm of χ computed with various methods relative to CCSD, ϵ_{χ}^{CCSD}

Mol	HF	MP2	LDA	KT3	M06	TPSS
AlH	0.834	0.682	2.213	0.453	3.786	0.135
BH	2.713	1.652	3.381	0.378	2.705	0.284
BeH-	1.653	1.210	2.872	3.255		0.666
CH+	5.226	2.376	4.804	0.680	2.845	0.013
CO	0.158	0.122	0.156	0.121	0.484	0.177
FNO	0.838	2.653		1.505		3.802
H2	0.019	0.007	0.082	0.015	0.222	0.007
H2O	0.081	0.080	0.136	0.023	0.629	0.029
H2S	0.104	0.183	0.235	0.111	1.110	0.084
HF	0.080	0.049	0.110	0.012	0.506	0.051
HOF	0.236	0.063	0.336	0.351	0.609	0.261
LiF	0.116	0.061	0.139	0.121	0.541	0.109
LiH	0.073	0.048	0.171	0.218	1.235	0.007
N2	0.144	0.110	0.179	0.143	0.381	0.222
NH3	0.058	0.105	0.177	0.067	0.727	0.027
SiH+	0.655	0.532	1.834	0.449	2.359	0.244

Table 30: Error norm of χ computed with various methods relative to MP2, ϵ_{χ}^{MP2}

Mol	HF	LDA	KT3	M06	TPSS
AlF	0.079	0.222	0.216	0.772	0.223
AlH	0.163	1.532	0.229	3.115	0.547
BH	1.061	1.730	1.274	1.096	1.368
BeH-	0.443	1.759	2.075		1.099
C2H4	0.151	0.477	0.401	0.094	0.367
C3H4	0.335	0.489	0.732	0.618	0.573
C4H4	0.439	0.997	0.953	1.728	0.627
CH+	2.850	2.428	3.056	0.486	2.363
CH2O	0.397	0.974	0.285	1.379	0.349
CH3F	0.140	0.133	0.265	0.518	0.153
CH4	0.170	0.178	0.212	0.708	0.182
CO	0.279	0.267	0.242	0.406	0.297
FCCH	0.142	0.230	0.226	0.651	0.204
FCN	0.100	0.265	0.225	0.532	0.226
FNO	3.329		4.041		6.398
H2	0.012	0.076	0.010	0.216	0.004
H2C2O	0.234	0.189	0.421	0.773	0.368
H2O	0.161	0.056	0.100	0.549	0.056
H2O2	0.396	0.337	0.439	0.750	0.307
H2S	0.270	0.052	0.292	0.934	0.267
H4C2O	0.174	0.288	0.575	0.552	0.404
HCN	0.090	0.330	0.245	0.439	0.304
HCP	0.214	0.659	0.455	0.702	0.521
HF	0.128	0.062	0.041	0.457	0.008
HFCO	0.194	0.406	0.351	0.165	0.362
HOF	0.239	0.358	0.389	0.584	0.294
LiF	0.177	0.119	0.129	0.495	0.103
LiH	0.025	0.217	0.262	1.282	0.054
N2	0.251	0.289	0.253	0.375	0.332
N2O	0.051	0.267	0.398	0.579	0.308
NH3	0.163	0.073	0.171	0.623	0.129
OCS	0.114	0.383	0.479	0.707	0.401
OF2	0.454	0.686	0.613	0.430	0.507
PN	0.690	1.134	0.927	1.390	1.127
SO2	1.014	0.867	0.914	0.189	0.985
SiH+	0.124	1.303	0.088	1.831	0.289

Table 31: Error norm of $\mathcal{A}$ computed with various methods relative to CCSD, $\epsilon_{\mathcal{A}}^{CCSD}$

Mol	HF	MP2	LDA	KT3	M06	TPSS
AlH	1.663	0.573	0.952	0.996	6.167	0.514
BH	0.222	0.226	0.542	0.800	3.814	0.374
BeH-	11.158	12.528	95.842	108.252	60.307	
CH+	0.210	0.058	0.167	0.147	0.356	0.016
CO	0.261	0.300	0.286	0.246	2.572	0.122
FNO	45.368	8.147		43.082		123.645
H2	0.034	0.012	0.221	0.130	0.685	0.066
H2O	0.209	0.145	0.262	0.207	1.183	0.192
H2S	0.022	0.214	0.356	0.308	3.581	0.123
HF	0.153	0.085	0.207	0.181	0.811	0.172
HOF	0.324	0.144	1.221	0.439	1.406	0.552
LiF	0.315	0.193	0.383	0.347	1.531	0.324
LiH	0.188	0.145	1.164	0.903	5.689	0.212
N2	0.107	0.137	0.209	0.165	2.272	0.073
NH3	0.230	0.189	0.372	0.296	1.921	0.216
SiH+	0.368	0.144	0.070	0.044	1.053	0.052

Table 32: Error norm of $\mathcal{A}$ computed with various methods relative to MP2, $\epsilon_{\mathcal{A}}^{MP2}$

Mol	HF	LDA	KT3	M06	TPSS
AlF	0.497	0.254	0.231	5.060	0.235
AlH	1.107	0.397	0.423	5.598	0.100
BH	0.045	0.322	0.582	3.589	0.149
BeH-	1.485	83.317	95.800		47.781
C2H4	0.250	0.170	0.341	3.377	0.386
C3H4	0.396	0.302	1.239	2.751	0.844
C4H4	0.803	0.980	1.492	4.271	1.239
CH+	0.151	0.109	0.206	0.412	0.070
CH2O	0.416	0.175	0.330	2.135	0.187
CH3F	0.147	0.135	0.479	1.269	0.301
CH4	0.206	0.230	0.100	1.716	0.090
CO	0.561	0.050	0.063	2.273	0.180
FCCH	0.248	0.147	0.362	4.507	0.420
FCN	0.284	0.460	0.505	2.855	0.535
FNO	37.425		35.122		115.569
H2	0.023	0.209	0.119	0.674	0.055
H2C2O	0.399	0.429	0.999	4.032	0.951
H2O	0.353	0.120	0.088	1.040	0.053
H2O2	0.360	0.910	0.535	1.022	0.469
H2S	0.220	0.175	0.206	3.367	0.127
H4C2O	0.380	0.456	1.072	1.881	0.662
HCN	0.277	0.081	0.074	2.485	0.138
HCP	0.461	0.212	0.487	4.932	0.756
HF	0.238	0.121	0.095	0.725	0.087
HFCO	0.564	0.135	0.387	3.134	0.303
HOF	0.320	1.102	0.322	1.274	0.436
LiF	0.507	0.202	0.220	1.354	0.164
LiH	0.076	1.304	1.047	5.827	0.358
N2	0.218	0.084	0.063	2.139	0.069
N2O	0.302	0.299	0.490	2.947	0.435
NH3	0.413	0.190	0.193	1.734	0.072
OCS	0.437	0.869	0.989	4.060	0.962
OF2	0.656	2.095	0.711	2.668	0.884
PN	0.758	0.490	0.565	4.067	0.839
SO2	0.786	0.694	1.134	4.376	1.075
SiH+	0.226	0.096	0.125	0.908	0.195

Table 33: Error norm of $\mathcal{M}$ computed with various methods relative to CCSD, $\epsilon_{\mathcal{M}}^{CCSD}$

Mol	HF	MP2	LDA	KT3	M06	TPSS
AlH	0.165	0.129	0.021	0.100	0.303	0.021
BH	0.535	0.246	0.591	0.177	0.158	0.000
BeH-	0.107	0.099	0.846	0.373		0.856
CH+	0.808	0.335	0.718	0.198	0.403	0.007
CO	0.200	0.121	0.019	0.037	0.214	0.016
FNO	7.061	8.526		3.407		8.362
H2	0.000	0.000	0.000	0.000	0.000	0.000
H2O	0.035	0.011	0.021	0.071	0.081	0.041
H2S	0.064	0.019	0.050	0.041	0.139	0.014
HF	0.024	0.004	0.016	0.041	0.062	0.027
HOF	0.300	0.026	0.144	0.075	0.139	0.069
LiF	0.024	0.021	0.044	0.033	0.409	0.035
LiH	0.082	0.033	0.044	0.160	0.277	0.081
N2	0.000	0.000	0.000	0.000	0.000	0.000
NH3	0.027	0.010	0.016	0.082	0.055	0.044
SiH+	0.143	0.110	0.040	0.014	0.107	0.052

Table 34: Error norm of $\mathcal{M}$ computed with various methods relative to MP2, $\epsilon_{\mathcal{M}}^{MP2}$

Mol	HF	LDA	KT3	M06	TPSS
AlF	0.194	0.044	0.003	0.232	0.030
AlH	0.037	0.150	0.028	0.174	0.107
BH	0.288	0.345	0.423	0.088	0.246
BeH-	0.206	0.747	0.274		0.757
C2H4	0.000	0.000	0.000	0.000	0.000
C3H4	0.161	0.149	0.060	0.162	0.061
C4H4	0.000	0.001	0.001	0.001	0.001
CH+	0.472	0.383	0.533	0.068	0.328
CH2O	0.151	0.050	0.013	0.342	0.028
CH3F	0.055	0.008	0.014	0.124	0.014
CH4	0.000	0.000	0.000	0.000	0.000
CO	0.321	0.102	0.083	0.093	0.105
FCCH	0.062	0.030	0.089	0.561	0.031
FCN	0.057	0.139	0.038	0.216	0.072
FNO	15.418		5.258		1.057
H2	0.000	0.000	0.000	0.000	0.000
H2C2O	0.200	0.115	0.127	0.553	0.138
H2O	0.045	0.011	0.060	0.071	0.031
H2O2	0.076	0.064	0.074	0.142	0.044
H2S	0.081	0.033	0.031	0.123	0.017
H4C2O	0.215	0.087	0.042	0.554	0.021
HCN	0.023	0.089	0.014	0.091	0.034
HCP	0.045	0.023	0.146	0.123	0.158
HF	0.028	0.011	0.037	0.058	0.023
HFCO	0.123	0.080	0.072	0.215	0.061
HOF	0.325	0.162	0.079	0.147	0.084
LiF	0.045	0.023	0.011	0.387	0.014
LiH	0.049	0.076	0.192	0.310	0.113
N2	0.000	0.000	0.000	0.000	0.000
N2O	0.074	0.018	0.054	0.139	0.008
NH3	0.037	0.006	0.072	0.045	0.034
OCS	0.006	0.034	0.034	0.703	0.021
OF2	0.114	0.428	0.195	0.464	0.225
PN	0.192	0.270	0.255	0.371	0.354
SO2	0.017	0.231	0.233	0.286	0.257
SiH+	0.033	0.071	0.124	0.003	0.163
